# Kidney-specific WNK1 amplifies kidney tubule responsiveness to potassium via WNK body condensates

**DOI:** 10.1172/JCI188792

**Published:** 2025-06-10

**Authors:** Cary R. Boyd-Shiwarski, Rebecca T. Beacham, Jared A. Lashway, Katherine E. Querry, Shawn E. Griffiths, Daniel J. Shiwarski, Sophia A. Knoell, Nga H. Nguyen, Lubika J. Nkashama, Melissa N. Valladares, Anagha Bandaru, Allison L. Marciszyn, Jonathan Franks, Mara Sullivan, Simon C. Watkins, Aylin R. Rodan, Chou-Long Huang, Sean D. Stocker, Ossama B. Kashlan, Arohan R. Subramanya

**Affiliations:** 1 Department of Medicine, Renal-Electrolyte Division, University of Pittsburgh, Pittsburgh, Pennsylvania, USA.; 2 Pittsburgh Center for Kidney Research, University of Pittsburgh School of Medicine, Pittsburgh, Pennsylvania, USA.; 3 Department of Medicine, Vascular Medicine Institute,; 4 Department of Bioengineering, Swanson School of Engineering,; 5 Center for Biological Imaging, and; 6 Department of Cell Biology, University of Pittsburgh, Pittsburgh, Pennsylvania, USA.; 7 Department of Internal Medicine, Division of Nephrology and Hypertension,; 8 Department of Human Genetics, and; 9 Molecular Medicine Program, University of Utah, Salt Lake City, Utah, USA.; 10 Medical Service, VA Salt Lake City Healthcare System, Salt Lake City, Utah, USA.; 11 Department of Internal Medicine, Division of Nephrology and Hypertension, University of Iowa Carver College of Medicine, Iowa City, Iowa, USA.; 12 Department of Neurobiology and; 13 Department of Computational and Systems Biology, University of Pittsburgh, Pittsburgh, Pennsylvania, USA.; 14 VA Pittsburgh Healthcare System, Pittsburgh, Pennsylvania, USA.

**Keywords:** Cell biology, Nephrology, Epithelial transport of ions and water, Molecular biology, Protein kinases

## Abstract

To maintain potassium homeostasis, the kidney’s distal convoluted tubule (DCT) evolved to convert small changes in blood [K^+^] into robust effects on salt reabsorption. This process requires NaCl cotransporter (NCC) activation by the with-no-lysine (WNK) kinases. During hypokalemia, the kidney-specific WNK1 isoform (KS-WNK1) scaffolds the DCT-expressed WNK signaling pathway within biomolecular condensates of unknown function termed WNK bodies. Here, we show that KS-WNK1 amplified kidney tubule reactivity to blood [K^+^], in part via WNK bodies. In genetically modified mice, targeted condensate disruption trapped the WNK pathway, causing renal salt wasting that was more pronounced in females. In humans, WNK bodies accumulated as plasma potassium fell below 4.0 mmol/L, suggesting that the human DCT experiences the stress of potassium deficiency, even when [K^+^] is in the low-to-normal range. These data identify WNK bodies as kinase signal amplifiers that mediate tubular [K^+^] responsiveness, nephron sexual dimorphism, and BP salt sensitivity. Our results illustrate how biomolecular condensate specialization can optimize a mammalian physiologic stress response that impacts human health.

## Abstract 2

KS-WNK1 is a kidney-specific scaffolding protein that organizes other proteins within biomolecular condensates called WNK bodies—structures essential for maintaining blood potassium and human health.

## Graphical abstract

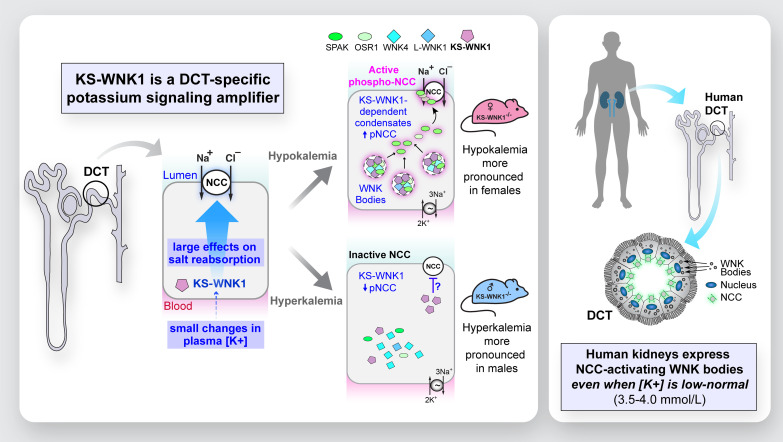

## Introduction

During environmental stress, physiologic systems must sense imbalance and coordinate appropriately tuned responses that maintain homeostasis. An example of this is the distal nephron, a series of kidney tubule segments that sense and cooperatively maintain plasma potassium concentrations within the narrow physiologic window required for life (1). During the stress of hypokalemia, a serine-threonine kinase cascade within the distal convoluted tubule (DCT) activates the thiazide-sensitive NaCl cotransporter (NCC; *SLC12A3*) via phosphorylation (2). Hypokalemia-mediated NCC activation limits downstream delivery of sodium to the connecting tubule and collecting duct, diminishing distal voltage-dependent potassium secretion. This minimizes urinary cation losses to conserve total body potassium (3). In contrast, hyperkalemia inhibits NCC, facilitating kaliuresis (4).

With-no-lysine (WNK) kinases are essential regulators of NCC phosphorylation and potassium homeostasis. During hypokalemia, the WNKs activate the kinases STE20/SPS1-related proline-alanine-rich protein kinase (SPAK, also known as *STK39*) and OSR1 (*OXSR1*), which phosphorylate NCC directly (1). Conversely, hyperkalemia promotes NCC dephosphorylation (4, 5). The integration of these signals generates an inverse relationship between NCC phosphorylation status and plasma [K^+^] (6). The importance of WNK signaling in potassium metabolism is evidenced by familial hyperkalemic hypertension, a Mendelian syndrome caused by overactivation of the DCT-expressed WNK signaling pathway, resulting in NCC hyperphosphorylation, salt-sensitive hypertension, and hyperkalemia that is cured with thiazide diuretics (7, 8).

**Figure 1:**
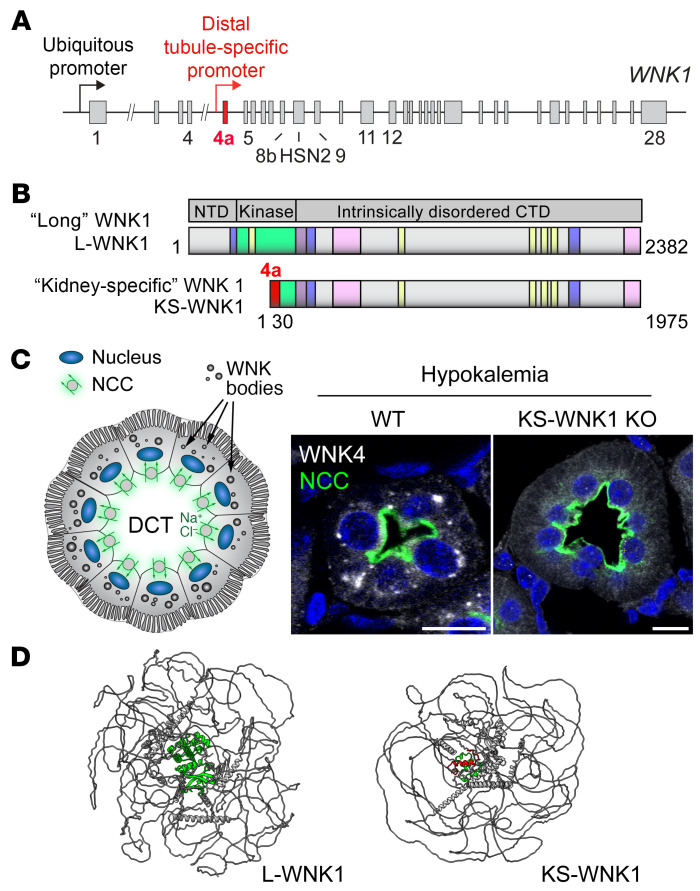
KS-WNK1 is a DCT-specific disordered protein that drives WNK body condensate formation. (**A**) Schematic representation of the *WNK1* gene. A ubiquitously expressed promoter drives L-WNK1 transcription. A distal tubule–specific promoter drives the expression of the truncated KS-WNK1 isoform. Figure numbers represent exons 1-28. (**B**) Domain architecture of L- and KS-WNK1 isoforms. Full-length long WNK1 contains an N-terminal domain (NTD), a serine-threonine kinase domain (green), and a >100 kDa intrinsically disordered C-terminal domain (CTD) that drives condensate formation (17). Other functional signatures include SPAK/OSR1 binding motifs (yellow), coiled-coil domains (purple), and prion-like regions (pink). KS-WNK1 lacks the NTD and most of the kinase domain, which was replaced by a 30-amino acid sequence encoded by exon 4a (red). Figure numbers represent amino acids (amino acid 1-2382 for L-WNK1 and amino acid 1-1975 for KS-WNK1). Schematics adapted with permission from Boyd-Shiwarski et al. (12). (**C**) Illustration (left) and immunofluorescence (IF) staining (right) of WNK bodies, which form in NCC-expressing DCT cells during hypokalemia. KS-WNK1–KO mice fail to form WNK bodies. Scale bar: 15 μm. (**D**) Alphafold3 predictions of L-WNK1 and KS-WNK1, highlighting their extensive disorder. The green structured region in L-WNK1 is the kinase domain, and the red structured region in KS-WNK1 is exon 4a.

The DCT features a unique complement of WNK-SPAK/OSR1 pathway gene products. WNK4 is the dominant DCT-expressed WNK kinase (9). The full-length kinase-active long isoform of WNK1 (L-WNK1) is also expressed in this nephron segment, though its abundance is low (10). Instead, the most abundant WNK1 isoform in the DCT is a kidney-exclusive truncated gene product that lacks kinase activity. This kidney-specific WNK1 (KS-WNK1) isoform requires an intragenic promoter located within intron 4 of the *WNK1* gene (Figure 1A). This distal tubule–specific promoter drives the expression of an alternative first exon that replaces the L-WNK1 N-terminus and most of the kinase domain with 30 unique amino acids encoded by exon 4a (11). Downstream from this sequence, L-WNK1 and KS-WNK1 are identical (Figure 1B). Exon 4a emerged during the evolutionary transition of vertebrates from water to land, a process that required robust kidney tubular transport to preserve electrolyte homeostasis (12, 13). Exon 4a is also critical for the formation of WNK signaling puncta during hypokalemia. These KS-WNK1–dependent foci, termed WNK bodies, influence the spatial localization of the DCT WNK-SPAK/OSR1 pathway (12) (Figure 1C). Hypokalemic WNK bodies contain WNK4, L-WNK1, SPAK, and OSR1, and their appearance correlates with NCC activation (12, 14, 15), suggesting that KS-WNK1’s role in WNK body formation is linked to sodium transport.

WNK bodies are micron-sized spheroid non-membrane-bound low-density regions of the cytoplasm that fail to colocalize with conventional organelle markers (12, 14, 16) (Figure 1). Thus, they are biomolecular condensates — membraneless cytosolic foci that assemble via phase transitions (17, 18). Though KS-WNK1–dependent WNK bodies are exclusive to the distal nephron, WNK kinases such as L-WNK1 and WNK3 are more ubiquitously expressed, and their ability to form condensates is a fundamental property that allows them to control ion transport and cell volume in many cell types (17, 19). For example, during acute hypertonic cell shrinkage, L-WNK1, SPAK, and OSR1 undergo crowding-induced phase separation, causing their rapid activation within cytosolic liquid-like droplets. Following activation, SPAK and OSR1 leave these dynamic structures and accumulate at the plasma membrane to phosphorylate NKCC1 (*SLC12A2*) and KCCs (*SLC12A4-7*), ubiquitously expressed NCC-like cotransporters that coordinate cell volume recovery. L-WNK1–dependent phase separation is mediated by its large C-terminal domain, a > 100 kDa intrinsically disordered region (IDR) that efficiently condenses in response to crowding (17) (Figure 1B). Notably, the truncated KS-WNK1 isoform lacks intrinsic kinase activity but contains the entire disordered C-terminal domain; in fact, this large phase separation-driving IDR comprises greater than 90% of the entire KS-WNK1 protein (Figure 1, B and D). This strongly suggests that KS-WNK1 functions as an intrinsically disordered scaffold that coordinates WNK4-SPAK/OSR1 activity in the DCT via condensed phase signaling (20).

Though KS-WNK1 mediates distal tubule WNK body assembly, its physiologic role in NCC regulation remains unresolved. Here, we report that KS-WNK1 functions as an amplifier of the WNK signaling pathway that operates across the entire physiologic range of [K^+^], allowing the DCT to optimize NCC-mediated salt reabsorption in response to potassium imbalance. By employing mouse models of KS-WNK1 absence and dysfunction, we demonstrate that this effect is WNK body dependent, establishing a previously unrecognized role for biomolecular condensates in mammalian potassium and BP homeostasis. We further show that the effect is sex specific, as females require WNK body–mediated signaling to amplify DCT salt reabsorption during hypokalemia. Finally, we show that, in the human kidney, WNK body expression progressively increases at a [K^+^] below 4.0 mmol/L, suggesting condensate-mediated activation of tubular salt reabsorption when potassium concentrations are within the low-to-normal reference range. This suggests a role for WNK bodies in human salt-sensitive hypertension. Together, our results identify WNK bodies as kidney-specific signaling condensates that regulate BP and potassium homeostasis.

## Results

### KS-WNK1 amplifies the inverse relationship between NCC phosphorylation and blood [K^+^] via multiple mechanisms.

**Figure 2:**
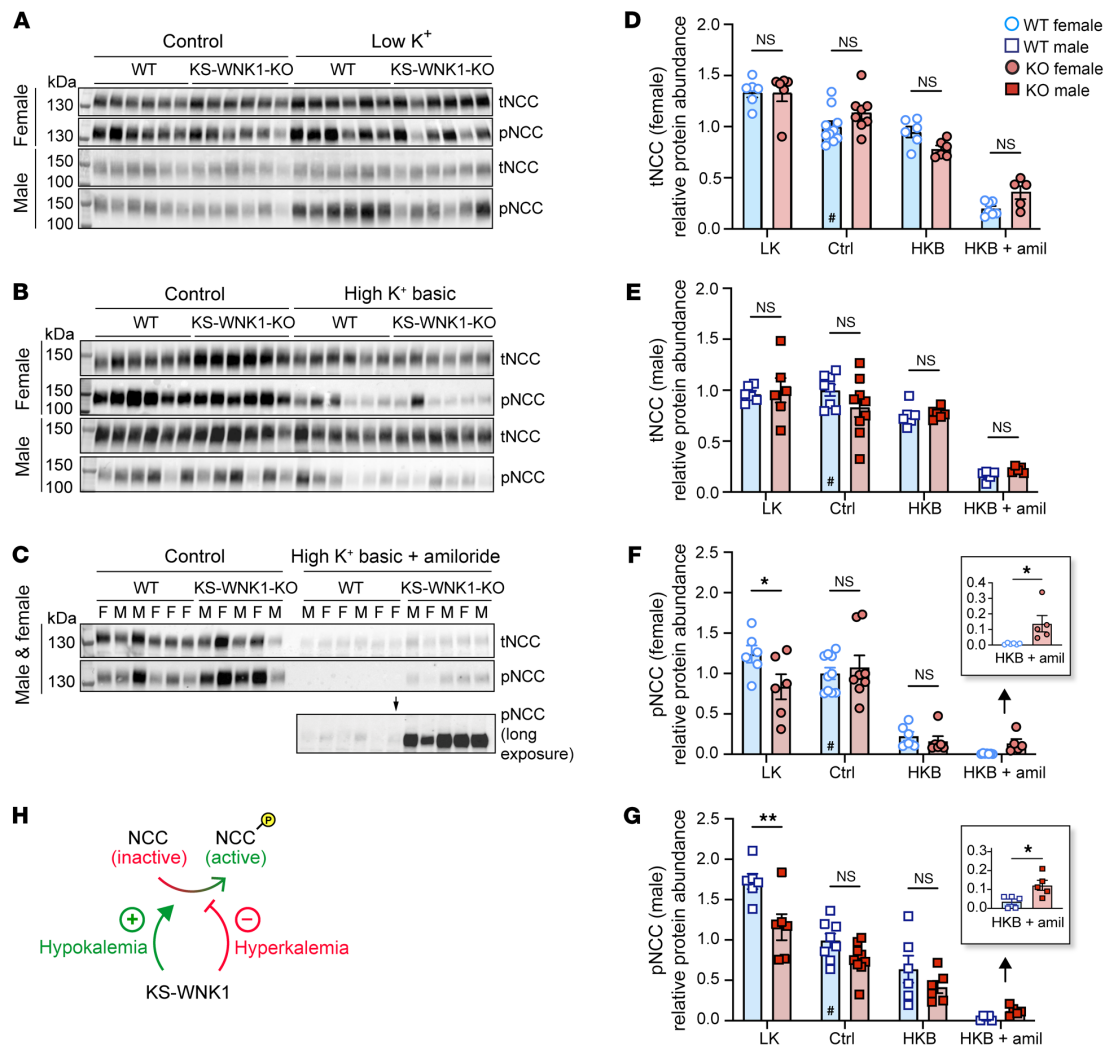
KS-WNK1 differentially alters NCC abundance and phosphorylation during hypo- and hyperkalemia. (**A**–**C**) Immunoblot analysis of kidney cortical extracts from female and male WT littermates and KS-WNK1–KO mice subjected to 10-day maneuvers that alter K^+^ homeostasis. Immunoblots of total NCC (tNCC) and active phospho-Thr53 NCC (pNCC), from female and male mice fed control diet (Ctrl) or (**A**) low K^+^ (LK), (**B**) high K^+^ basic (HKB), or (**C**) HKB + amiloride (HKB + amil) (2 mg/kg/d) diet. In **A** and **B**, lanes corresponding to WT and KO animals on control diet were from replicate lysates to facilitate normalization between blots. The average from these replicates was plotted for control diet (Supplemental Figure 2). Additional values for WT and KO mice on control diet were obtained from Figure 2C. (**D** and **E**) KS-WNK1 had no significant effect on tNCC abundance, regardless of sex. (**F** and **G**) KS-WNK1 had significant effects on pNCC during LK and HKB+amiloride treatments. (**H**) These data indicate that KS-WNK1 stimulates NCC during hypokalemia and inhibits during hyperkalemia. Results are shown as mean ± SEM; *n* = 5–6 mice per genotype, sex, and diet, except control diet *n* = 8–10 mice. M, male; F, female. Two-way ANOVA with Šídák’s multiple comparisons test. *P ≤ 0.05, **P ≤ 0.01. For **D**–**G**, data were normalized to WT mice on control diet, as indicated by #.

To begin to understand the role of KS-WNK1–dependent WNK bodies in NCC regulation, we studied the effect of KS-WNK1 deletion on NCC expression and phosphorylation status in mice across a broad range of blood potassium concentrations. KS-WNK1–KO mice and WT littermates were administered diets with low K^+^ (LK), control, or alkaline high K^+^ (high K^+^ basic [HKB]) content for 10 days (Supplemental Table 1; supplemental material available online with this article; https://doi.org/10.1172/JCI188792DS1). Because K^+^-loaded mice with a normal glomerular filtration rate efficiently excrete a potassium load (1, 21), we also studied a cohort of HKB-fed mice supplemented with the potassium-sparing diuretic amiloride (2 mg/kg/d) to induce frank hyperkalemia (Supplemental Table 2). We then performed immunoblots for total NCC (tNCC) and phospho-Thr53 NCC (pNCC), a signature of NCC activation (Figure 2, A–C; Supplemental Figure 1, A–F; Supplemental Figure 2, A–H; and Supplemental Figure 3, A–D).

We initially compared NCC densitometry in WT and KS-WNK1–KO mice stratified by sex and dietary K^+^ manipulation. While this analysis did not reveal a significant effect of KS-WNK1 deletion on tNCC protein abundance (Figure 2, D and E), KS-WNK1–KO mice exhibited lower pNCC abundance during K^+^ restriction and higher pNCC abundance during K^+^ loading with amiloride (Figure 2, F and G). The effects of HKB plus amiloride on pNCC were confirmed with antibodies that recognize alternative NCC phosphoactivation sites at threonine-58 and serine-71 (Supplemental Figure 3B) (22). KS-WNK1–KO mice also exhibited higher pNCC abundance than WT controls following 10 days of potassium loading on a high KCl (5% potassium) diet (Supplemental Figure 4, A–C), which was sufficient to induce frank hyperkalemia in the absence of amiloride (Supplemental Figure 4, D and E). Thus, the effects of KS-WNK1 on pNCC during hyperkalemia were consistent across different physiologic manipulations. The requirement of KS-WNK1 expression for K^+^-induced inhibition of pNCC is supported by prior publications (23, 24). Collectively, these data indicate that KS-WNK1 exerts divergent effects on pNCC depending on blood potassium status. During hypokalemia, KS-WNK1 appears to be an activator of NCC, but during hyperkalemia, it appears to be an NCC inhibitor (Figure 2H). These findings comport with those of a recent report (23) and suggest that KS-WNK1 exerts complex effects on NCC activity that span the full physiologic spectrum of blood [K^+^].

**Figure 3:**
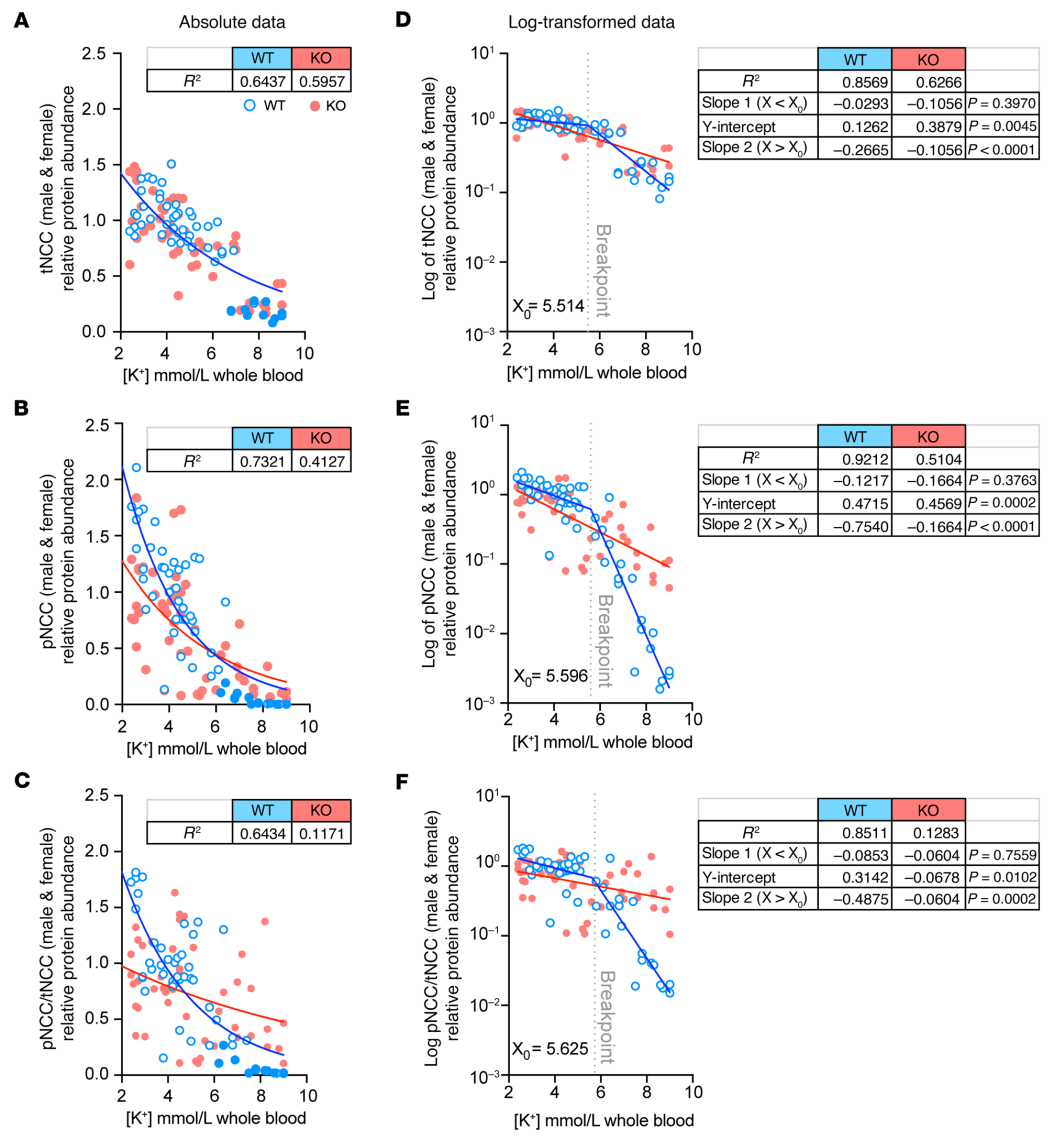
KS-WNK1 amplifies the inverse relationship between NCC phosphorylation and blood [K^+^]. Total and phosphorylated NCC protein abundance in KS-WNK1–KO (red) versus WT (blue) mice, plotted as a function of blood [K^+^]. (**A**–**C**) tNCC, pNCC, and pNCC /tNCC ratio, fit to single exponential curves. R^2^ measures are presented in table format alongside the graphs. For all graphs, the single exponential function adequately fit the WT data at [K^+^] <4.0 but overestimated data points at [K^+^] >6.0 (filled blue circles). (**D**–**F**) Normalized tNCC, pNCC, and pNCC/tNCC densitometry in **A**–**C** was log transformed and analyzed by linear regression. In all cases, WT data were best fit by a segmented linear regression regime, with *X*_0_ breakpoints (dotted line) around 5.6 mmol/L. Slopes of the 2 linear components are presented in table format alongside the corresponding graphs. For KO mice, slopes 1 (*X* < *X*_0_) and 2 (*X* > *X*_0_) did not differ as the log-transformed data were best fit by simple linear regression. *P* values represent slope comparisons between WT and KO data; since slope 1 comparisons did not reach significance, *Y*-intercept comparisons with *P* values are shown. See also Supplemental Figure 5 for results disaggregated by sex and residual plots.

To further explore these findings, we performed a regression analysis, plotting individual tNCC and pNCC densitometry values for mice subjected to the LK, control, HKB, and HKB plus amiloride treatments, as a function of blood potassium concentration measured at the time of sacrifice (Figure 3, A–C). For WT mice, these graphs revealed a steep increase in pNCC abundance below a potassium concentration of 4 mmol/L, indicating a nonlinear relationship between NCC phosphorylation and blood [K^+^]. This amplification effect was evident in the pNCC and pNCC/tNCC graphs and was clearly blunted in KS-WNK1–KO mice (Figure 3, B and C). We first attempted to fit these data to single exponential curves, but this model was inadequate, as it overestimated nearly all the measured NCC densitometry values at a blood [K^+^] >6 mmol/L for WT mice (Figure 3, A–C; note WT filled blue circles that consistently fall below the curve). Thus, to stabilize variance in NCC signal across measured blood [K^+^] and better visualize goodness of fit, we log transformed the densitometry data (Figure 3, D–F). As predicted by the suboptimal single exponential fits to the untransformed data, simple linear regression of these log transformations resulted in nonrandom residuals for WT mice, strongly suggesting that additional components are required to adequately model the observed NCC densitometry dependence on blood [K^+^] (Supplemental Figure 5A).

Subsequent curve-fitting trials revealed that the WT log-transformed data were best explained by a segmental linear regression model, which added 2 parameters: a second slope and a breakpoint dividing the segments (*X*_0_; Figure 3, D–F). For WT mice, the addition of a second linear component yielded symmetric and randomly distributed residuals, significantly improving the fit (*P* ≤ 0.0001 vs. straight line by *F* test for all 3 WT curves [tNCC, pNCC, and pNCC/tNCC]) (Supplemental Figure 5A). Remarkably, the tNCC, pNCC, and pNCC/tNCC breakpoints consistently settled at a blood [K^+^] of around 5.6 mmol/L; i.e., near the upper limit of the currently defined normal reference range for serum (25). In contrast to those in WT mice, the tNCC, pNCC, and pNCC/tNCC data in KS-WNK1–KO mice were best fit by simple linear regression (Figure 3, D–F), as goodness of fit was not improved by segmental linear regression. Furthermore, simple linear regression yielded symmetric and randomly distributed residuals for the KO data (Supplemental Figure 5B). These results indicate that while both WT and KS-WNK1–KO mice exhibit an inverse relationship between NCC and blood [K^+^], the WT mice require an additional component to account for the relationship between NCC and blood [K^+^] during hyperkalemia. These findings were concordant in males and females when the pNCC data were disaggregated by sex (Supplemental Figure 5, C–F).

This regression analysis uncovered key differences in the relationship between blood potassium and pNCC in WT and KS-WNK1–KO mice. At a [K^+^] of less than 5.6 mmol/L (i.e., *X* < *X*_0_), the log-transformed WT and KO slopes of pNCC and pNCC/tNCC were not different, but the *Y* intercepts for pNCC and pNCC/tNCC were significantly lower in KS-WNK1–KO mice (*P* = 0.0002 and 0.0102 for pNCC and pNCC/tNCC respectively; Figure 3, E and F). Thus, when blood potassium is <5.6 mmol/L, NCC phosphorylation status increased exponentially in both WT and KS-WNK1–KO mice as plasma K^+^ got progressively lower, an amplification effect that was significantly blunted in mice lacking KS-WNK1. At a blood potassium greater than 5.6 mmol/L (*X* > *X*_0_), the inverse relationship between [K^+^] and pNCC increased dramatically in WT mice, resulting in a marked negative deflection in slope (Figure 3, E and F, and Supplemental Figure 5, D and F). This suggests that WT mice recruit an auxiliary process that further suppresses NCC phosphorylation when blood [K^+^] >5.6 mmol/L. Because this deflection in slope is absent in KO mice, the auxiliary dephosphorylation mechanism appears to be KS-WNK1 dependent.

Collectively, these data suggest that KS-WNK1 steepens the inverse relationship between blood [K^+^] and NCC activation through discrete mechanisms that differentially affect NCC phosphorylation and dephosphorylation. In other words, KS-WNK1 expands the dynamic range of NCC phosphorylation status in response to changes in blood [K^+^], converting small changes in plasma potassium into large effects on pNCC abundance.

### Relationship between blood [K^+^] and WNK body expression.

**Figure 4:**
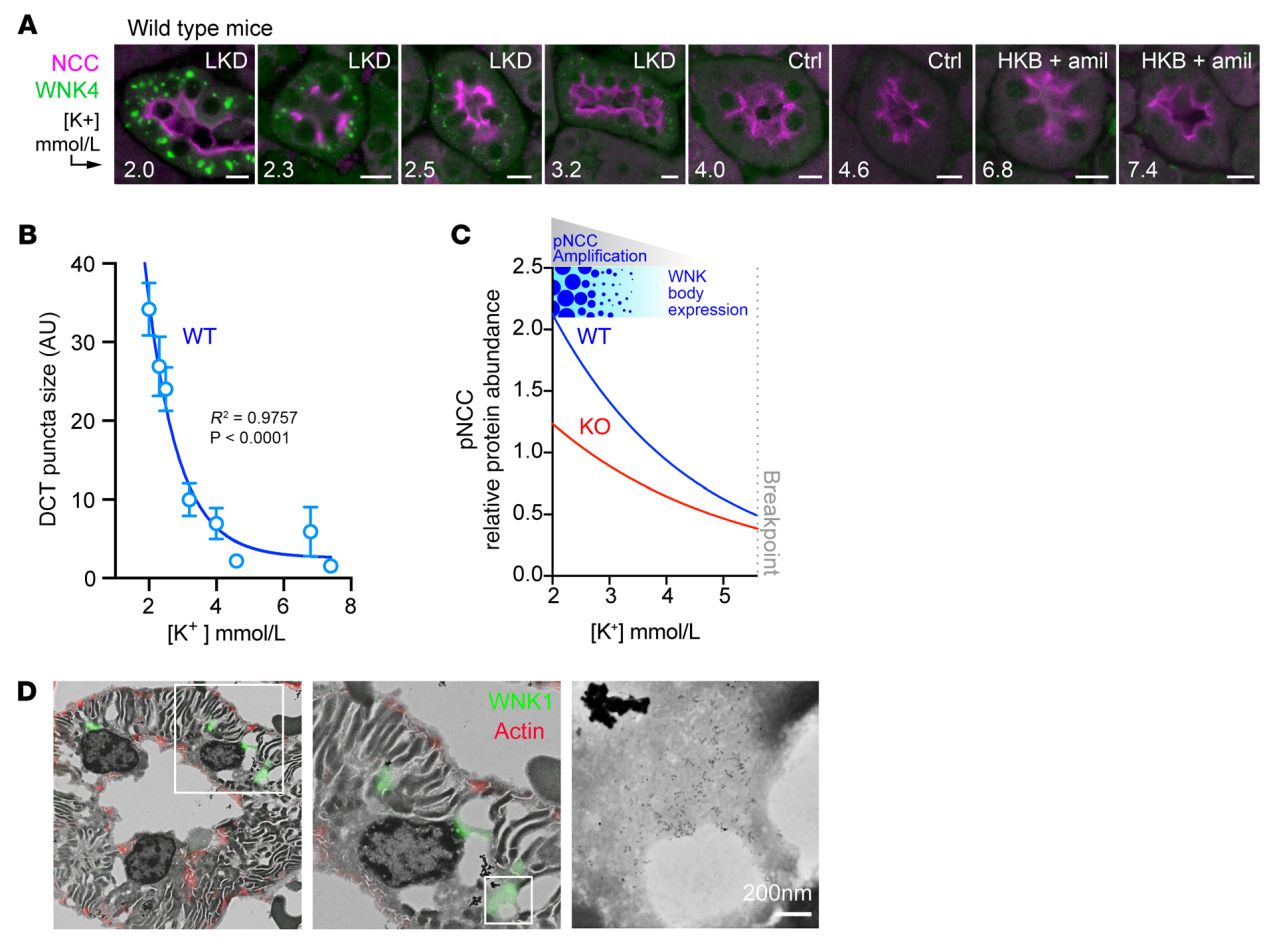
WNK body condensate expression is dependent upon blood [K^+^] and correlates with pNCC amplification during potassium deficiency. (**A**) IF of WNK bodies in WT male mice treated with various potassium maneuvers for 10 days to induce a broad range of blood K^+^ concentrations. DCTs were identified by NCC costaining. WNK4^+^ puncta progressively increased in size as [K^+^] fell and were not visible above a [K^+^] of 4.0. Scale bar: 10 μm (**B**) Quantification of WNK body size as a function of blood [K^+^], fit to a single exponential curve; *R*^2^ = 0.9757, *P* < 0.0001 vs. a horizontal line through the mean of Y values. This demonstrates a WNK body size dependence on [K^+^]. (**C**) Cropped and adapted image from Figure 3B integrated with WNK body expression and pNCC amplification. As [K^+^] falls below 4.0 mmol/L, WT mice amplify NCC phosphorylation more effectively than KS-WNK1–KO mice, correlating with WNK body expression. (**D**) CLEM of a semithin (~300 nm) DCT section in a hypokalemic WT mouse combining confocal with backscattered-electron scanning electron microscopy (BSE-SEM). WNK bodies were detected with a WNK1 primary and a dual Alexa Fluor 488/5 nm gold particle–conjugated secondary. The image is inverted; thus, areas of low signal intensity represent lower BSE reflectivity. WNK body condensates contained immunogold signal that clustered within membraneless perinuclear cytosolic regions of lower material density. Scale bar: 200 nm.

We hypothesized that the potassium-dependent changes in NCC phosphorylation status correspond with altered WNK body expression. To test this, we performed DCT immunostaining for WNK bodies in kidneys harvested from WT male mice with a broad range of blood potassium concentrations. As blood potassium levels progressively decreased below 4 mmol/L, WNK body size increased exponentially (Figure 4, A and B). By contrast, WNK bodies were not visible in WT males with a plasma potassium greater than 4.0 mmol/L. Thus, similar to the effects of hypokalemia on pNCC abundance, WNK body expression correlates inversely with changes in blood [K^+^] (Figure 4C). This suggests that the nucleation and growth of these condensates is linked to the amplification of NCC activity during potassium deficiency.

We have previously published that in an in vitro cellular model exogenous WNK bodies have distinct ultrastructures that are electron hypodense, membraneless assemblies that do not colocalize with conventional organelle markers (12). Using a correlative light and electron microscopy (CLEM) method that combines confocal microscopy with backscattered electron detection via scanning electron microscopy (SEM) on semithin kidney sections revealed that endogenous WNK bodies have similar properties (Figure 4D).

### KS-WNK1 increases WNK-SPAK/OSR1 pathway abundance during K^+^ deficiency but does not influence its abundance during K^+^ excess.

**Figure 5:**
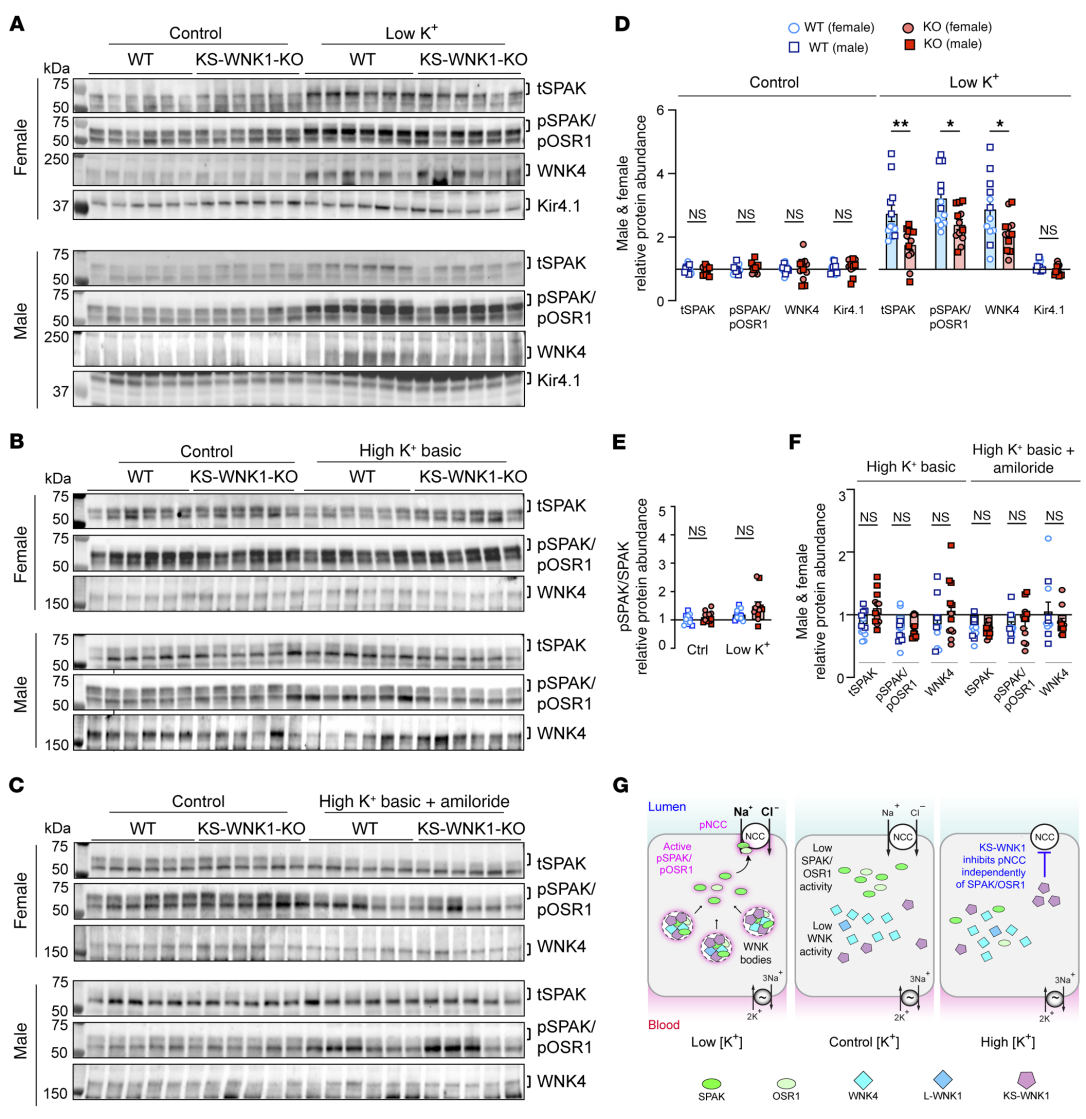
Dysregulated WNK4-SPAK/OSR1 pathway activity in KS-WNK1–KO mice during K+ restriction, but not during K^+^ loading. Immunoblot analysis of kidney cortical extracts from female and male WT littermates and KS-WNK1–KO mice subjected to various K^+^ maneuvers for 10 days. (**A**–**C**) Immunoblot of the WNK-SPAK/OSR1 pathway from mice treated with control diet or (**A**) low K^+^ diet, (**B**) HKB diet, or (**C**) HKB + amiloride. Brackets indicate the band analyzed. In **A**–**C**, lanes corresponding to WT and KO animals on control diet were from replicate lysates to facilitate normalization between blots. The values graphed for control diet in Figures **D** and **E** represent an average of the replicates (Supplemental Figure 6). (**D**) WT mice fed a low K^+^ diet had significant increases in tSPAK, pSPAK/pOSR1, and WNK4 compared with WT mice on control diet. KS-WNK1–KO mice had a blunted response to the low K^+^ diet compared with WT mice. (**E**) Phosphorylated-to-total SPAK ratio in WT and KO mice subjected to control vs. low K^+^ diet. (**F**) No differences in WNK4-SPAK/OSR1 pathway abundance or phosphorylation in WT and KS-WNK1–KO mice subjected to HKB or HKB + amiloride treatment. (**G**) WNK-SPAK/OSR1 pathway activation during low, control, and high blood [K^+^] experimental maneuvers. During low [K^+^], KS-WNK1–dependent WNK bodies condense the WNK-SPAK/OSR1 pathway; this correlates with SPAK/OSR1 and NCC phosphoactivation. During high K^+^, KS-WNK1 inhibits pNCC activation independently of the SPAK/OSR1 pathway. Results are shown as mean ± SEM; *n* = 12 mice per genotype and diet (males and females combined). Two-way ANOVA with Šídák’s multiple comparisons test, *P ≤ 0.05, **P ≤ 0.01.

The DCT-expressed WNK4-SPAK/OSR1 pathway constitutes the canonical NCC activation signal (8). Given the role of WNK bodies in controlling its localization, we interrogated this signaling cascade in KS-WNK1–KO mice. Consistent with prior reports (2, 26), K^+^ restriction in WT mice upregulated the expression of WNK4, total SPAK (tSPAK), and phospho-Ser (pSer) 373 SPAK/pSer 325 OSR1 relative to control diets (Figure 5, A and D). By comparison, K^+^-restricted KS-WNK1–KO mice exhibited weaker WNK4-SPAK/OSR1 pathway upregulation (Figure 5, A and D). We did not observe a difference in pSPAK normalized to tSPAK in WT and KO mice on LK, contrary to prior reports (23) (Figure 5E). In contrast to the LK maneuver, WT and KO mice exhibited no differences in WNK4, tSPAK, or pSPAK/pOSR1 expression in the context of HKB or HKB plus amiloride treatments that increase blood [K^+^] (Figure 5, B, C, and F). This supports findings by Penton et al. who demonstrated that high K^+^ directly and rapidly controls NCC phosphorylation independent of the SPAK/OSR1 pathway (27). Thus, the ability of KS-WNK1 to amplify NCC phosphorylation during K^+^ deficiency correlates with increased WNK4-SPAK/OSR1 signaling via WNK bodies, but its ability to augment NCC dephosphorylation during K^+^ excess does not (Figure 5G).

### KS-WNK1 and WNK body localization.

**Figure 6:**
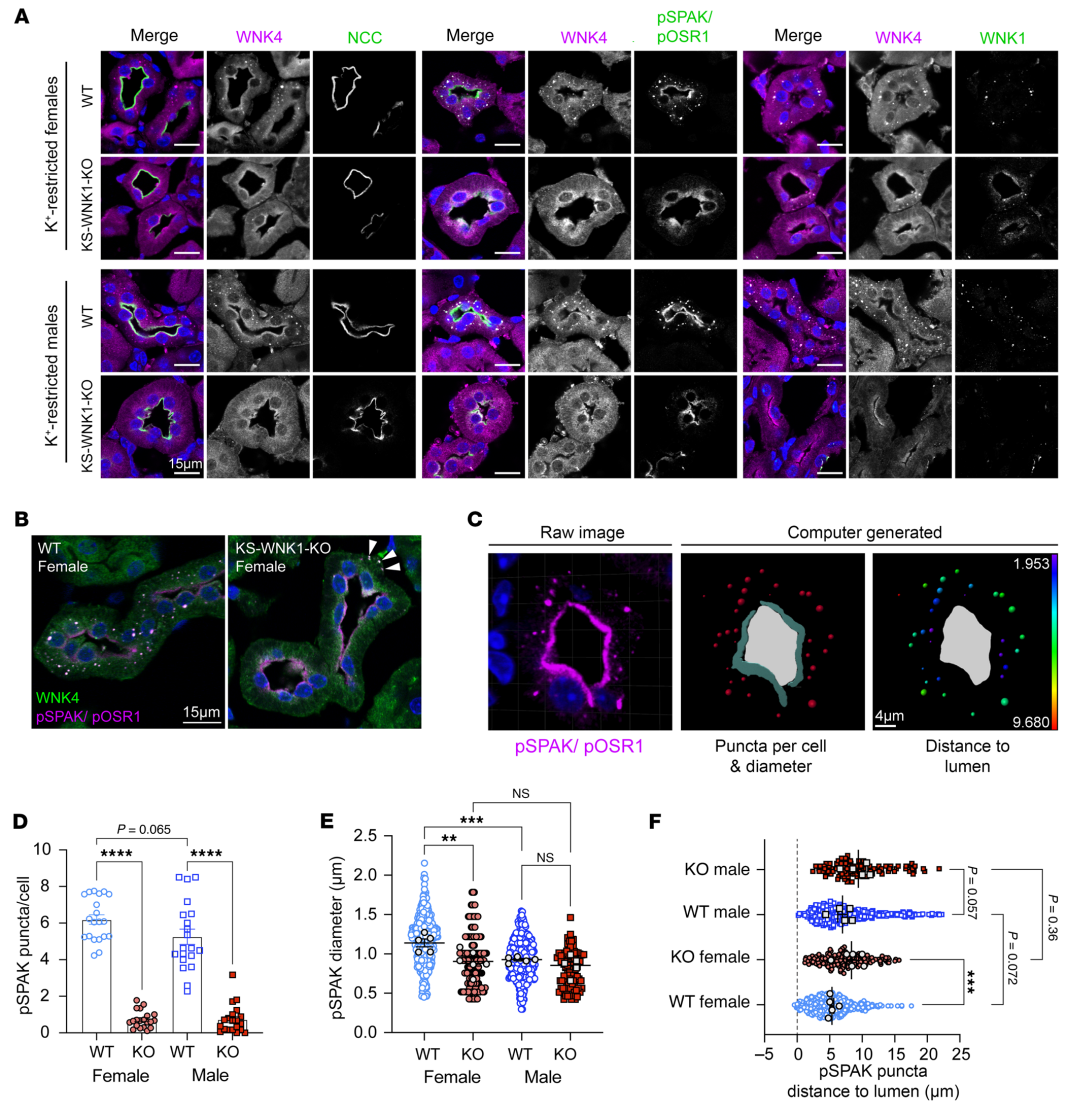
K^+^-restricted WT and KS-WNK1–KO mice exhibit sex differences in WNK body expression. (**A**) IF of kidney sections from WT or KS-WNK1–KO mice treated with low K^+^ diet for 10 days. DCTs were identified by NCC costaining and morphology. WNK4, pSPAK/pOSR1, and WNK1 antibodies colocalized within puncta in WT mice, whereas puncta were nearly absent in KS-WNK1–KO mice (duplicate bottom left image with Figure 1C). Scale bars: 15 μm. (**B**) WNK body formation in female WT and KS-WNK1–KO mice. Cytosolic puncta are largely absent in KS-WNK1–KO mice, though pSPAK/pOSR1 apical staining is present. Rarely, mislocalized basolateral puncta containing pSPAK/pOSR1 and WNK4 were observed (arrowheads). Scale bar: 15 μm. (**C**) Imaris was used to quantify WNK body number and size (middle) and distance to lumen (right) from raw confocal IF images of pSPAK/pOSR1 puncta (left). Scale bar: 4 μm. (**D**–**F**) Quantification of pSPAK/pOSR1. (**D**) Puncta per cell (20 tubules per condition), (**E**) puncta diameter (5 tubules per condition), and (**F**) distance to apical lumen in female and male mice (5 tubules per condition). Two-way ANOVA with Šídák’s multiple comparison, *P ≤ 0.05, **P ≤ 0.01, ***P ≤ 0.001, ****P ≤ 0.0001.

Because the effect of KS-WNK1 on WNK-SPAK/OSR1 signaling likely predominates when hypokalemia-induced WNK bodies are present (12), we evaluated the effects of KS-WNK1 deletion on WNK-SPAK/OSR1 localization during dietary K^+^ restriction. Consistent with our prior report (12), K^+^ restriction was associated with the formation of DCT-specific WNK bodies that stained positive for WNK1, WNK4, and pSPAK/pOSR1, suggesting that WNK4 activates its downstream targets within condensates (Figure 6A). These structures were largely absent in potassium-deficient KS-WNK1–KO mice regardless of sex (Figure 6, A and D). Even so, pSPAK/pOSR1 apical staining was present despite KS-WNK1 deletion (Figure 6B), indicating that KS-WNK1 is not necessary for active SPAK to engage with NCC during hypokalemia. However, given the observation that KS-WNK1–KO mice exhibited blunted SPAK/OSR1 and NCC phosphorylation during K^+^ deficiency (Figure 2, A and G; Figure 3, B and E; and Figure 5, A and D), the data indicate that KS-WNK1 potentiates SPAK/OSR1 activation in response to decreased blood [K^+^], likely through WNK body–mediated signaling.

As reported previously (12), KS-WNK1–KO DCT cells rarely exhibited WNK-SPAK/OSR1^+^ puncta that were mislocalized to the basal pole during K^+^ restriction (Figure 6B, arrowheads). Morphometric analysis (Figure 6C) revealed that while both sexes exhibited a comparable number of WNK bodies per cell during K^+^ restriction (Figure 6D), females exhibited larger condensates that were positioned closer to the tubular lumen (Figure 6, E and F). These findings suggest sex-specific differences in WNK-SPAK/OSR1 pathway functionality.

### Sex-specific effects of KS-WNK1 on blood and urine electrolytes.

**Figure 7:**
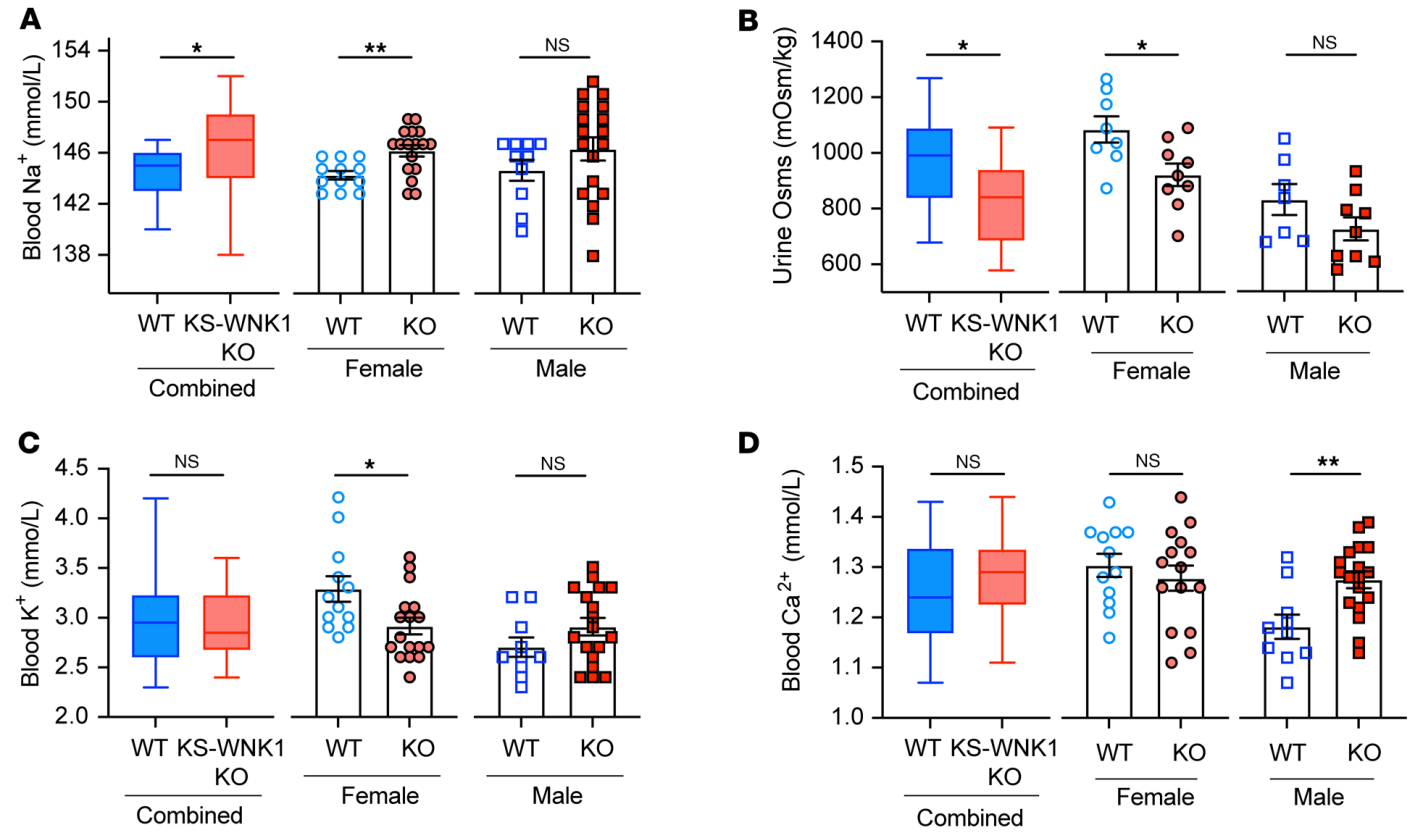
Effect of KS-WNK1 on blood and urine composition in K^+^-restricted female and male mice. Male and female whole blood electrolytes and urine were obtained from WT and KS-WNK1–KO mice fed low K^+^ diets for 10 days. Results were analyzed as both combined and sex-disaggregated. (**A**) KS-WNK1–KO mice had significantly increased blood [Na^+^] when males and females were analyzed in combination and when females were analyzed separately. (**B**) Urine osmolality in KS-WNK1–KO mice was significantly decreased in the combined male and female dataset and in the female pool. Mean urine osmolality in KS-WNK1–KO males was lower than in male WT mice, without reaching significance. (**C**) KS-WNK1 deletion had no significant effect on whole blood [K^+^] in the combined pool or in the male pool. However, female KS-WNK1–KO mice had a significant decrease in whole blood [K^+^] compared with sex-matched controls. (**D**) KS-WNK1 deletion had no significant effect on whole blood [Ca^2+^] in the combined male and female pool or in the female pool. However, male KS-WNK1–KO mice had a significant increase in whole blood [Ca^2+^]. Sample size: *n* = 10–18 mice. Unpaired *t* test between WT and KO was used to determine significance, *P ≤ 0.05, **P ≤ 0.01.

We also observed diverging trends between WT and KS-WNK1–KO mice when blood and urine data were disaggregated by sex. Sex-dependent differences were more evident at the extremes of low and high K^+^ and included effects on blood K^+^, Cl^–^, HCO_3_^–^, Ca^2+^, and urine osmolality and pH. When placed on a K^+^-deficient diet, where NCC phosphorylation was high and KS-WNK1 dependent (Figure 2), KS-WNK1–KO females exhibited more pronounced hypernatremia, hypokalemia, and reduced urine osmolality compared with WT females (Figure 7, A–C, and Supplemental Tables 2 and 3). We did not observe differences in urine K^+^, as these measurements were at the low limit of detection on a K^+^-deficient diet (Supplemental Table 3). In contrast to females, KS-WNK1–KO males did not exhibit lower blood K^+^ versus WT males during potassium restriction (Figure 7C), despite strong effects of KS-WNK1 deletion on pNCC abundance (Figure 2). Male KS-WNK1–KO mice also did not exhibit differences in blood Na^+^ or urine osmolality. Relative to WT littermates, however, male KS-WNK1–KO mice were more hypercalcemic during K^+^ restriction (Figure 7D and Supplemental Table 2). Thus, K^+^-restricted KS-WNK1–KO mice exhibit features commonly seen in states of low NCC activity — low blood potassium levels and higher blood calcium levels — similar to Gitelman syndrome or thiazide diuretic administration (8).

Next, we investigated whether the relative hypokalemia in K^+^-restricted KS-WNK1–KO females was due to increases in aldosterone (aldo), ENaC, or renal outer medullary potassium channel (ROMK) — known factors that can cause hypokalemia. During dietary K^+^ restriction, aldo levels tended to be equal to or lower in KS-WNK1–KO mice (Supplemental Table 2), consistent with prior observations (28). While it is conceivable that lower aldo levels observed in KS-WNK1–KO mice could be explained by increased ENaC activation, we observed the opposite, with lower expression of the uncleaved form of γENaC (Supplemental Figure 7, A–C) and could not detect expression of the subunit’s cleaved/active form in either WT or KO mice during dietary K^+^ restriction (Supplemental Figure 7, A–C). Collectively, these findings suggest that the hypokalemia in female KS-WNK1–KO mice is not due to increased aldo or ENaC, but instead reduced NCC activity. Others have reported that KS-WNK1–KO mice have decreased ENaC expression (28, 29), possibly to compensate for increased NCC activity. We also found that ROMK protein abundance was not elevated in KS-WNK1–KO female mice relative to WT sex-matched littermates (Supplemental Figure 7, D and E). While we did not observe KS-WNK1–induced changes in ROMK abundance, earlier in vitro studies suggest that KS-WNK1 promotes ROMK activation (30, 31). Mouse models have shown that KS-WNK1–KO mice exhibit reduced ROMK activity (24, 32), despite an increased localization of ROMK at the apical membrane in KS-WNK1–KO mice (28, 29). Further research is needed to understand how KS-WNK1 and ROMK interact and regulate serum potassium levels across the range of blood K^+^.

### Role of KS-WNK1 in BP regulation, salt sensitivity, and thiazide responsiveness.

**Figure 8:**
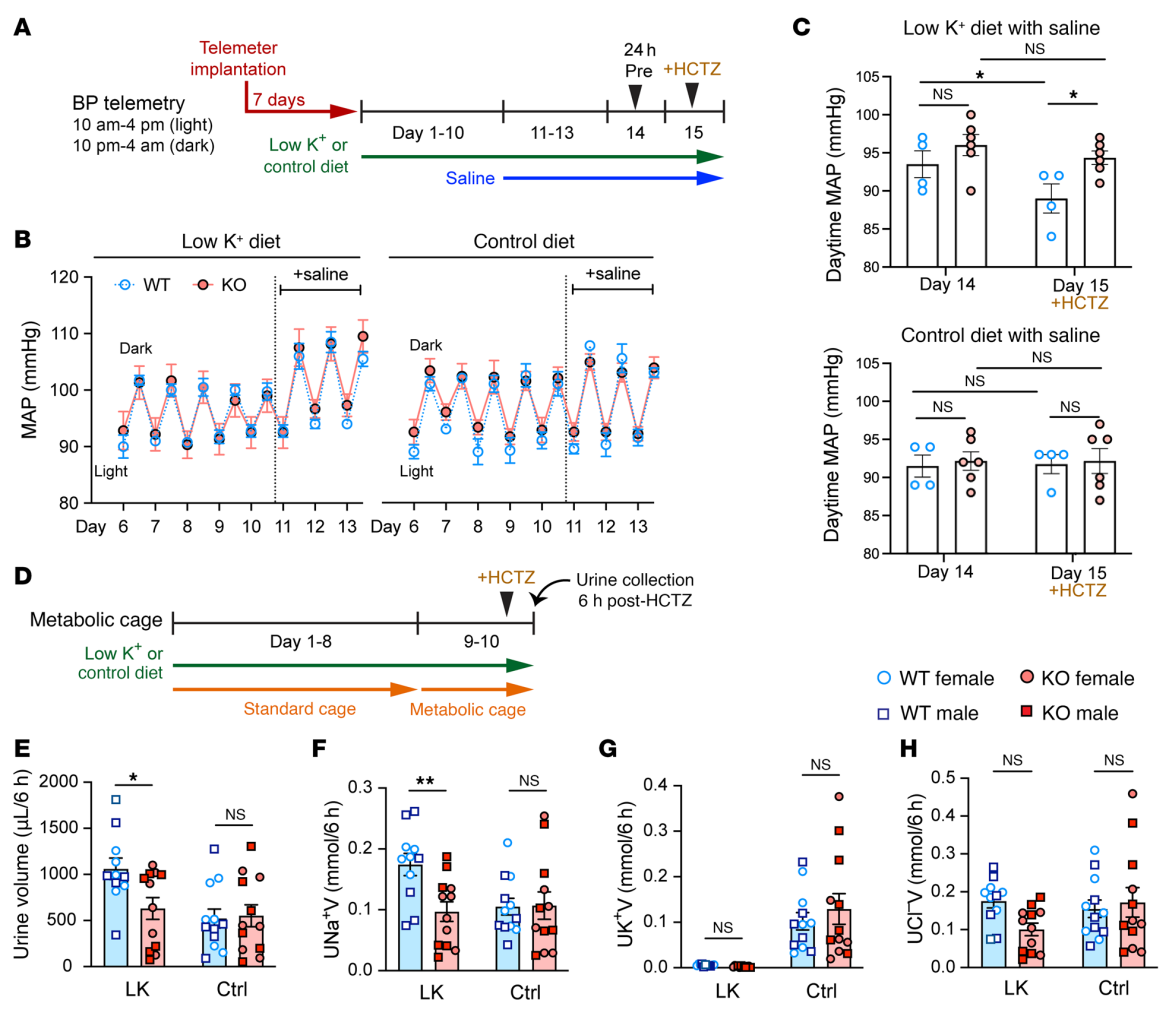
Potassium-restricted KS-WNK1–KO mice are thiazide insensitive. (**A**) Schematic of the BP telemetry experiment. Female WT (*n* = 4) and KS-WNK1–KO mice (*n* = 6) were subjected to either low K^+^ or control diets for 10 days, followed by supplementation with 1% normal saline in drinking water for 3 days and then hydrochlorothiazide (HCTZ) treatment. (**B**) KS-WNK1 expression had no significant effect on mean arterial pressure (MAP) in K^+^-restricted or control diet–fed mice. Saline supplementation increased MAP in K^+^-restricted mice. Genotype had no significant effect determined by 2-way ANOVA with post hoc Šídák’s test. Each data point represents either daytime or nighttime MAP averaged over 6 hours. (**C**) Thiazide challenge. Mice were fed low K^+^ or control diet for 10 days and then 1% saline in their drinking water for 3 days. Daytime MAP was measured for a 6-hour window, starting on day 14 (24 hours before HCTZ injection) and on day 15 (1 hour after HCTZ injection) (25 mg/kg IP). WT mice on low K^+^ diet had a significant decrease in MAP with HCTZ administration, compared with KS-WNK1–KO mice. *P ≤ 0.05, 2-way ANOVA with post hoc Šídák’s test. (**D**) Schematic of the metabolic cage experiment. Diuretic challenge was performed in both female and male WT and KS-WNK1–KO mice. After 10 days of control or low K^+^ diet, mice were injected with HCTZ (25 mg/kg IP), and urine was collected for 6 hours. (**E**) Urine volume and (**F**) urine Na^+^V were greater in WT mice compared with those in KO mice. (**G**) On the low K^+^ diet, urine K^+^ was too low to detect a significant difference. (**H**) There was a trend for HCTZ to blunt Cl^–^ excretion in KS-WNK1–KO mice on low K^+^ diet, without reaching significance. Results are shown as mean ± SEM; *n* = 12 mice per genotype and diet. Two-way ANOVA with Šídák’s post test; *P ≤ 0.05, **P ≤ 0.01. See also Supplemental Tables 2 and 3.

Given the importance of NCC in BP regulation (3), we performed telemetric BP measurements in KS-WNK1–KO mice (Figure 8A). These studies focused on female mice since they exhibited larger electrolyte differences during K^+^ restriction (Figure 7). Despite differences in pNCC expression (Figure 2), 10 days of potassium deprivation yielded no differences in mean arterial pressure (MAP) between WT and KS-WNK1–KO female mice (Figure 8B). Moreover, though K^+^-restricted WT and KO mice both developed a salt-sensitive increase in BP (21), we observed no differences between KO mice and WT littermates. Female mice administered a control diet for 10 days did not exhibit changes in BP, either at baseline or after saline loading, regardless of KS-WNK1 genotype (Figure 8B). To test for differences in NCC activity, we challenged WT and KS-WNK1–KO mice with an i.p. injection of hydrochlorothiazide (HCTZ; 25 mg/kg). WT mice on K^+^ restriction responded to HCTZ injection with a significant 4.5 mmHg average decrease in MAP. In contrast, KS-WNK1–KO mice were relatively insensitive to HCTZ (Figure 8C). Thus, similar to humans with Gitelman syndrome (33), K^+^-restricted KS-WNK1–KO female mice exhibit low NCC activity but are able to maintain their BP via compensatory effects.

To further assess the effects of KS-WNK1 on NCC activation, we administered HCTZ and measured urinary volume, Na^+^, K^+^, and Cl^–^ in male and female mice maintained on either a LK or control diet for 10 days (Figure 8D). K^+^-restricted KS-WNK1–KO mice exhibited a blunted response to HCTZ compared with WT littermates, with decreased urinary volume and UNa^+^V (urinary sodium multiplied by urinary volume) (Figure 8, E and F). There was no change in urinary K^+^, likely because it was near the lower limit of detection due to dietary K^+^ restriction (Figure 8G). There was also a trend for decreased urinary Cl^–^ under potassium-restricted conditions (Figure 8H). These diuretic and natriuretic responses reflect lower NCC activity in KS-WNK1–KO mice compared with WT control mice, consistent with the reduced NCC phosphorylation in KS-WNK1–KO mice after dietary K^+^ restriction (Figures 2 and 3). Interestingly, a previous study reported conflicting results, showing that KS-WNK1–KO mice exhibited increased thiazide-sensitivity on a control diet (24). These discrepancies could be due to differences in composition of the control diet or that the WT control mice in the prior study were not littermate controls.

### WNK bodies are required for NCC activation during hypokalemia.

**Figure 9:**
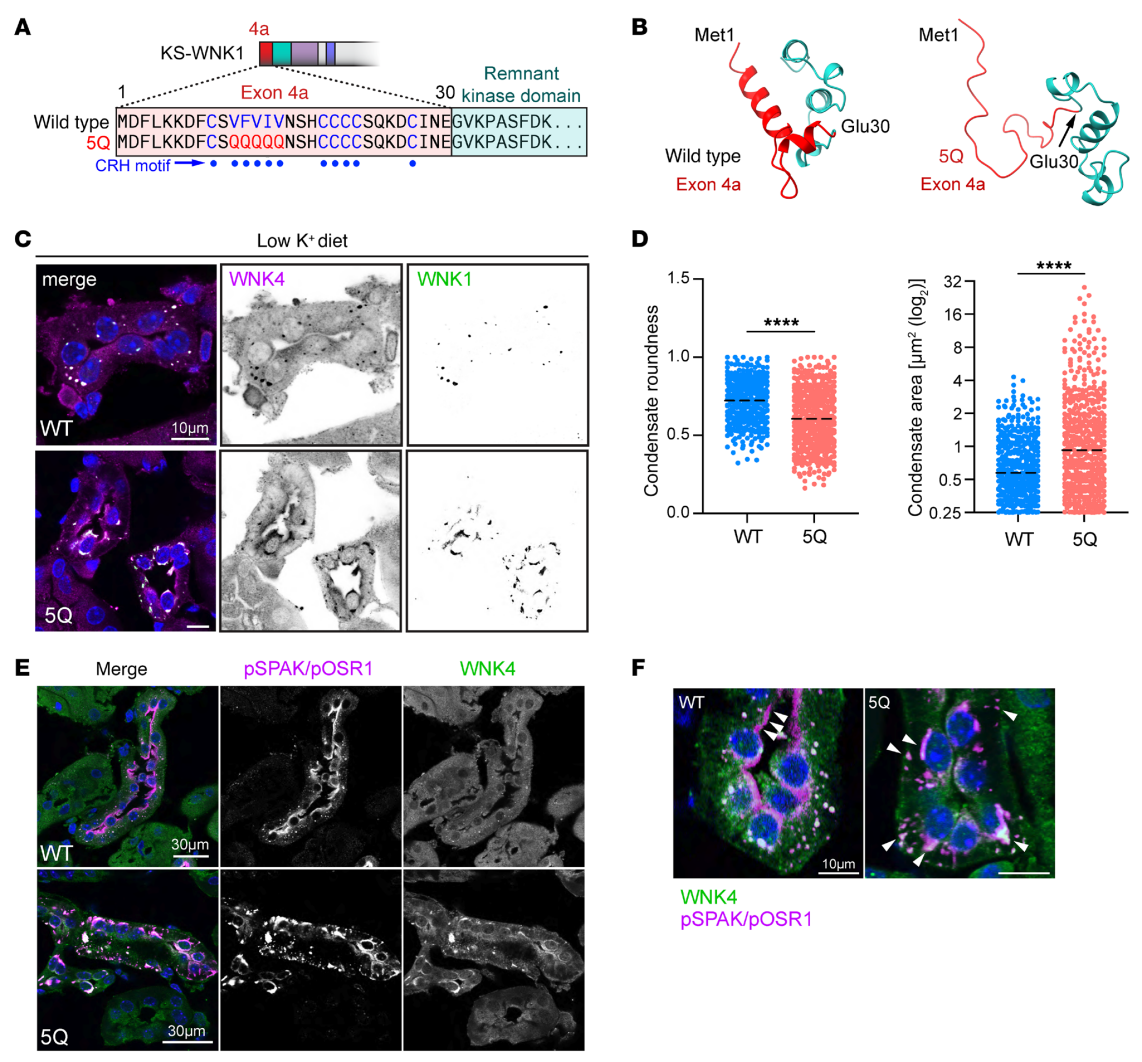
KS-WNK1 5Q mutant mice exhibit altered WNK body morphology and pSPAK localization. (**A**) Exon 4a of KS-WNK1 encodes a 30-amino acid sequence, including a cysteine-rich hydrophobic (CRH) motif. The motif’s 5 consecutive bulky hydrophobic residues were mutated to glutamines to generate “5Q” mice with aberrant WNK body formation. (**B**) AlphaFold predicted structures of the WT and 5Q exon 4a peptide (red) and the adjacent remnant kinase domain (cyan). The 5Q mutation disrupts a predicted helical structure encoded by exon 4a. (**C**) IF of kidneys from female 5Q mice maintained on low K^+^ diet for 10 days. Typical WNK bodies are absent and replaced by irregularly shaped foci that often form paranuclear crescents and contain WNK4 and pSPAK/pOSR1. Scale bar: 10 μm. (**D**) The morphology of the 5Q foci was less round and larger than WT WNK bodies. *n* = 425 foci from 9 images for WT, 530 from 12 images for 5Q; 2-tailed *t* test, ****P < 0.0001. (**E**) WNK4 and pSPAK/pOSR1 costaining in WT and 5Q mice. In WT mice, pSPAK/pOSR1 signal colocalized with WNK4 in puncta and was also located at the DCT apical membrane. In contrast, 5Q mice exhibited strong pSPAK/pOSR1 and WNK4 co-condensation in perinuclear aggregates but no apical pSPAK/pOSR1. Scale bar: 30 μm. (**F**) Higher magnification image of WNK4 and activated SPAK/OSR1 expression. White arrowheads highlight that, in WT mice, pSPAK/pOSR1 accumulates at the plasma membrane, but in 5Q mice, it becomes sequestered in irregularly shaped, generally subnuclear foci. Scale bar: 10 μm.

Our analyses in KO mice demonstrated that during potassium deficiency, KS-WNK1 drives WNK body formation (Figure 6), amplifies NCC phosphoactivation (Figure 3), and increases WNK4-SPAK/OSR1 pathway expression (Figure 5). However, these studies do not establish whether the blunted NCC activation in K^+^-restricted KS-WNK1–KO mice is specifically due to impaired WNK body condensation or other KS-WNK1–dependent factors. To address this, we generated a mouse expressing a full-length mutant version of KS-WNK1 that cannot form functional WNK bodies. As noted in Figure 1, the KS-WNK1 N-terminus is capped by 30 unique amino acids encoded by exon 4a. Previously, we showed that the exon 4a coding sequence contains a conserved cysteine-rich hydrophobic (CRH) motif that is necessary for WNK body formation in vitro (12). To disrupt WNK body formation in mice, we replaced 5 essential hydrophobic residues within the CRH motif with 5 neutral glutamines (VFVIV- > QQQQQ) (Figure 9A and Supplemental 8, A–D). AlphaFold predicted that this “5Q” mutation would disrupt the KS-WNK1 N-terminal structure, unraveling an amphipathic helix encoded by exon 4a (Figure 9B).

**Figure 10:**
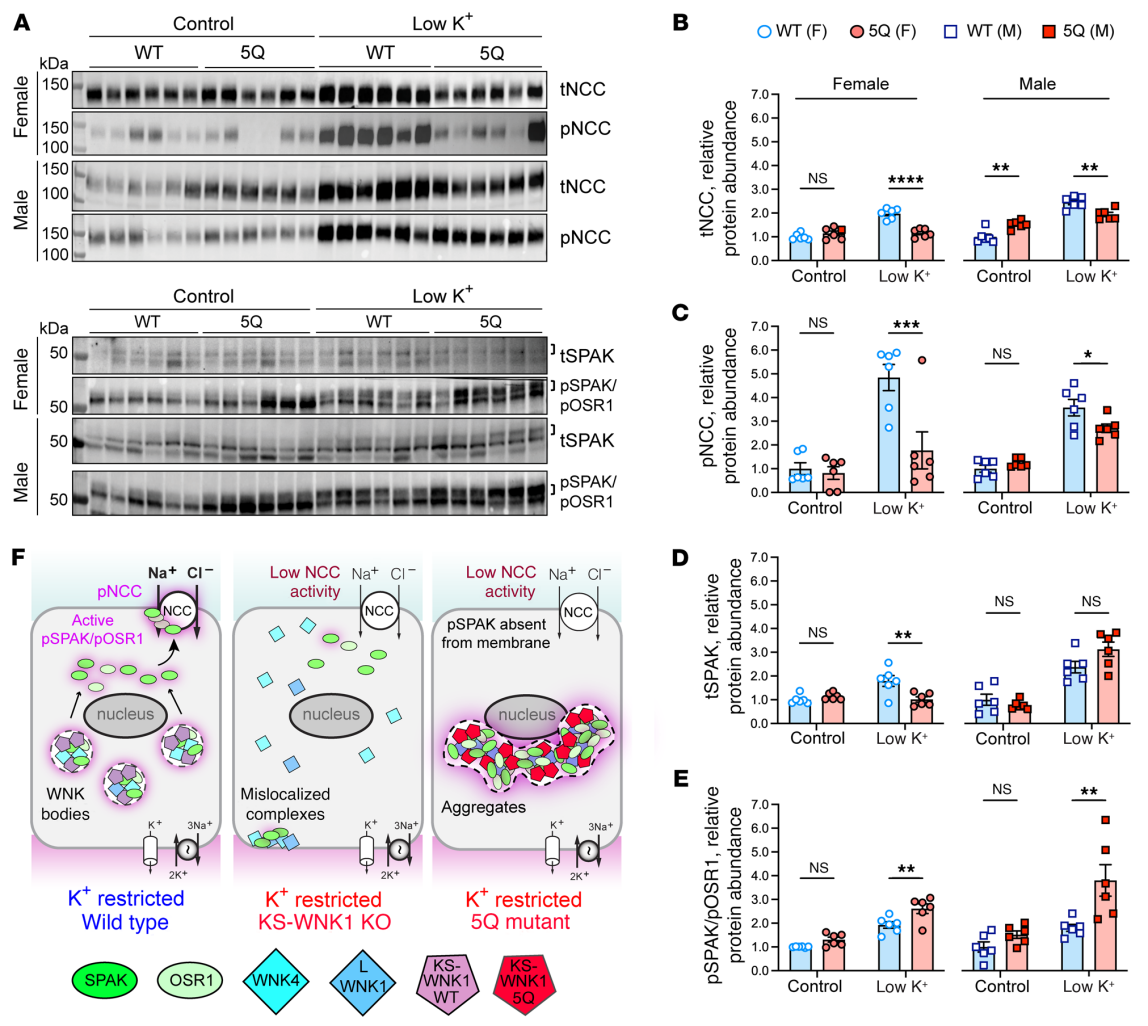
WNK bodies are necessary for KS-WNK1 to amplify NCC phosphorylation during hypokalemia. (**A**) WT and 5Q mice were fed control or low K^+^ diet for 10 days, and kidney cortex homogenates were probed for tNCC, pNCC, tSPAK, and pSPAK/pOSR1. Brackets indicate the band analyzed. (**B**–**E**) Graphical representation of immunoblots in **A**. (**B**) tNCC abundance. (**C**) pNCC abundance. (**D**) tSPAK abundance. (**E**) pSPAK/pOSR1 abundance. K^+^-restricted 5Q mice had significantly increased pSPAK/pOSR1 and reduced tNCC and pNCC expression, indicating that signaling to NCC was uncoupled. *n* = 6 mice per genotype, sex, and diet. Two-way ANOVA with Šídák’s post test was applied, *P < 0.05, **P < 0.01, ***P ≤ 0.001, ****P ≤ 0.0001. (**F**) Model of WNK-SPAK/OSR1-NCC signaling in WT, KS-WNK1–KO, and 5Q mice. KS-WNK1 normally facilitates WNK body condensate formation and NCC activation via the WNK-SPAK/OSR1 pathway. In K^+^-restricted KS-WNK1–KO mice, WNK bodies are largely absent and remaining complexes are mislocalized, resulting in low SPAK/OSR1 and NCC activity. In the K^+^-restricted 5Q mouse, WNK-pSPAK/pOSR1 becomes trapped in perinuclear aggregates, preventing pSPAK/pOSR1 expression at the DCT apical membrane, causing a reduction in NCC activity. See also Supplemental Table 4.

Mice homozygous for the KS-WNK1 5Q mutation were maintained on control or LK diet for 10 days and assessed for changes in SPAK/OSR1 and NCC activation by immunostaining, Western blot, and electrolyte measurements. Potassium-restricted 5Q mice were unable to form spheroid WNK bodies and instead formed larger amorphous aggregates with reduced roundness that accumulated around nuclei (Figure 9, C and D). These structures were enriched in pSPAK/pOSR1, which was absent from the apical membrane suggesting aberrant function (Figure 9, E and F). Consistent with intracellular sequestration and disruption of signaling, during K^+^ restriction pSPAK/pOSR1 abundance was increased in kidney immunoblots by 210% in males (*P* = 0.0019) and 136% in females (*P* = 0.0032), but pNCC abundance did not increase accordingly. Instead, pNCC decreased by 25% in males (*P* = 0.018) and 63% in females (*P* = 0.0008) (Figure 10, A–F; Supplemental Figure 9, A and B).

Similar to those in KS-WNK1–KO mice, the effects of potassium restriction were predominantly observed in females. Potassium-restricted female 5Q mice had a significantly decreased blood K^+^ and Cl^–^ and increased HCO_3_^–^, suggestive of a sex-specific Gitelman-like phenotype (Supplemental Table 4), whereas 5Q mice fed a control diet had an opposing effect, with males having the predominant phenotype exhibiting hyperchloremic metabolic acidosis (females trended toward this), with elevated Cl^–^ and lower HCO_3_^–^. Collectively, these findings indicate that the KS-WNK1 5Q mutation alters WNK body formation and function during K^+^ restriction, resulting in mislocalization of the WNK-SPAK/OSR1 pathway and low NCC activity.

### WNK body abundance correlates with serum [K^+^] in humans.

**Figure 11:**
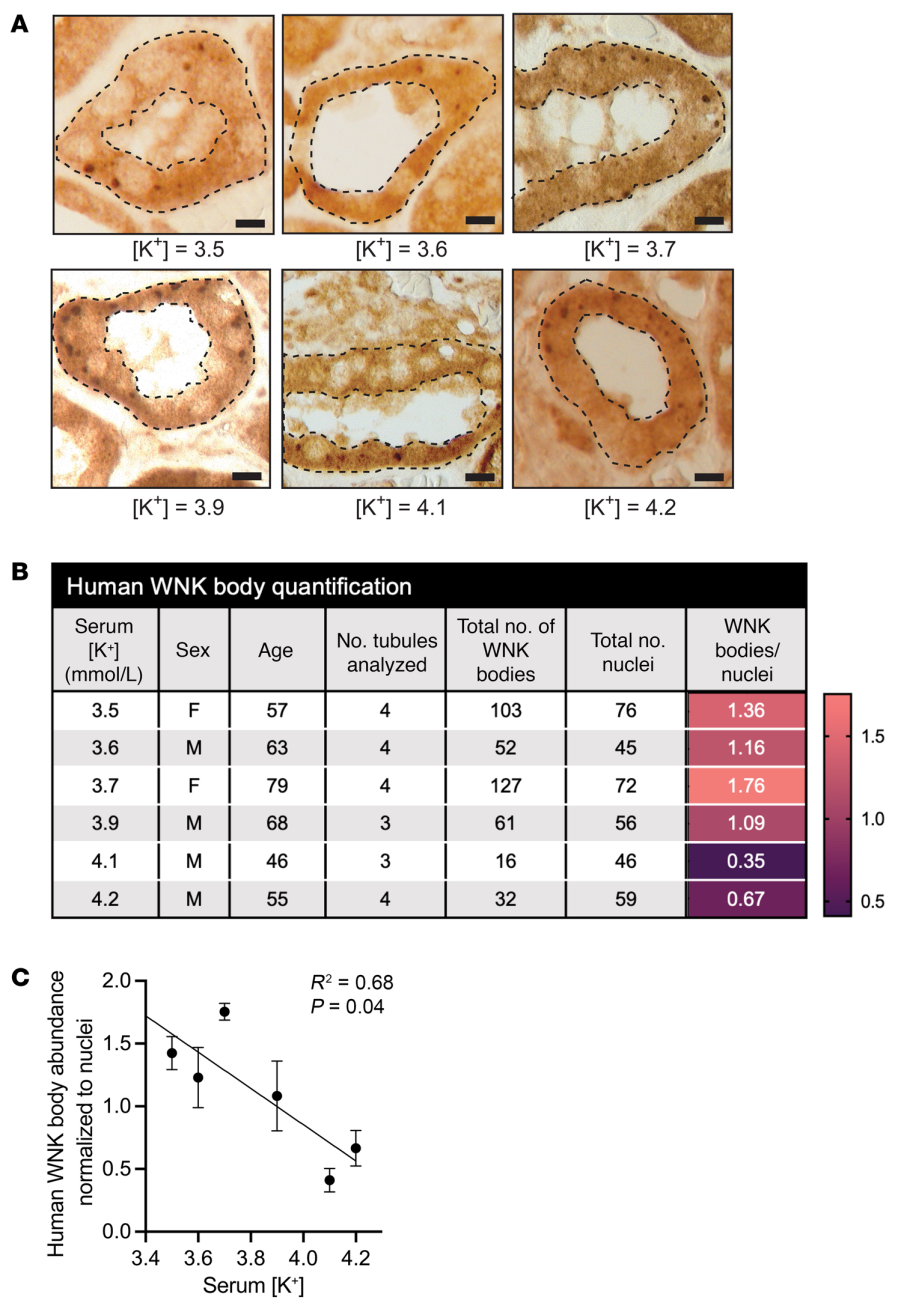
Human WNK body abundance correlates with serum [K^+^]. (**A**) Immunohistochemistry of DCTs obtained from 6 human kidney wedge biopsies stained for WNK1. DCTs were confirmed by NCC staining in adjacent sections (not shown). Scale bar: 10µm. (**B**) Serum [K^+^], sex, and age of the participants, along with the values used for quantification. A heatmap indicates the correlation between WNK bodies and increasing serum [K^+^]. (**C**) There was an inverse relationship between serum [K^+^] and WNK body abundance. To calculate the average number of WNK bodies per cell, the number of WNK bodies within a single tubule was counted and then normalized to the number of nuclei within that tubule. Each data point represents the average number of WNK bodies per kidney analyzed; *n* = 3–4 tubules analyzed per kidney. Slope of –1.446 calculated using simple linear regression. Results are shown as mean ± SEM; *r*^2^ = 0.68, *P* = 0.04 vs. horizontal line.

In WT mouse models, WNK bodies form during hypokalemia and disperse during normokalemia (12, 14) (Figure 4A). To date, human WNK bodies have only been reported in the setting of severe hypokalemic nephropathy, a pathologic condition caused by substantial K^+^ deficiency (16). To explore the physiological relevance of WNK bodies in human health, we asked whether these potassium-dependent condensates are present in humans with [K^+^] in the physiologic range (3.5–4.2 mmol/L). WNK bodies were present in all 6 kidney samples studied from male and female participants, ages 46–79 years (Figure 11A). Consistent with studies in mice, there was an inverse correlation between decreasing serum [K^+^] and increasing WNK body abundance (Figure 11, B and C). A progressive increase in WNK bodies was noted, particularly when the measured [K^+^] was <4.0 mmol/L (Figure 11, B and C).

## Discussion

First reported over a decade ago, WNK bodies were initially described as punctate clusters of the WNK-SPAK/OSR1 pathway that form in the DCT during potassium deficiency (15, 34). Subsequent studies noted that these foci are membraneless, consistent with the notion that they are specialized biomolecular condensates that assemble during potassium stress (12, 18). WNK body formation requires KS-WNK1, but the role of this isoform in DCT salt transport has been elusive. Early in vitro work reported that KS-WNK1 inhibits NCC-mediated sodium transport (35, 36). Subsequent studies in germline global KS-WNK1–KO mice by Hadchouel et al. (28) and Liu et al. (37) seemed to corroborate this, as baseline NCC phosphorylation was modestly increased in KO mice compared with controls. More recently, similar results were reported by Ferdaus et al. in a conditional DCT-specific KS-WNK1 KO (29). Despite these observations, other in vitro and in vivo studies have claimed the converse, that KS-WNK1 is an NCC activator (23, 38–40). To complicate matters further, Bahena-Lopez et al. recently proposed that KS-WNK1 can function as either an NCC inhibitor or activator depending on dietary potassium intake (23). These apparent contradictions are reconciled when the effect of KS-WNK1 on pNCC is analyzed as a function of blood [K^+^]. Subjecting over 100 KS-WNK1–KO mice and littermate controls to various potassium maneuvers designed to manipulate NCC phosphorylation across a wide physiologic range, we found that KS-WNK1–KO mice exhibited reduced pNCC during hypokalemia and increased pNCC relative to WT mice during hyperkalemia. Thus, KS-WNK1 can function as an activator or an inhibitor of NCC, depending on potassium status. Integrating these results as a function of blood [K^+^] in regression analyses, we found that the steep inverse relationship between NCC phosphorylation and potassium was blunted in KS-WNK1–KO mice. This indicates that KS-WNK1’s true function is to expand the dynamic range of NCC phosphorylation across the entire physiologic spectrum of blood potassium concentrations that are experienced during life. In order for the DCT to adjust salt reabsorption in response to potassium imbalance, it must sense small fluctuations in interstitial potassium concentrations and then convert those tiny changes into robust effects on NCC activity. Our findings demonstrate that KS-WNK1 is an essential part of this signal amplification mechanism.

The concept that KS-WNK1 amplifies the DCTs responsiveness to [K^+^] resolves the apparent discrepancies reported in prior studies of KS-WNK1–KO mice. All the in vivo experiments that reported an inhibitory effect of KS-WNK1 on NCC (23, 28, 29, 37) were conducted in mice with blood potassium concentrations that ranged between 4.33 and 7 mmol/L, while the experiment that reported an activating effect of KS-WNK1 on NCC (23) was carried out in mice with an average blood [K^+^] of 3.5 mmol/L. Given the flattened pNCC response curve observed in KS-WNK1–KO mice (Figure 3, B and E), blood potassium concentrations at the higher end of the spectrum would be associated with KS-WNK1–dependent inhibition, while potassium concentrations at the lower end would be associated with KS-WNK1–mediated activation. Thus, we conclude that the studies revealing KS-WNK1–KO mice as having slightly higher pNCC expression compared with WT mice were carried out at a point on the potassium response curve that favors mild KS-WNK1–dependent NCC inhibition. Deleting KS-WNK1 in this physiologic range would be insufficient to cause familial hyperkalemic hypertension. Slight discrepancies in the potassium response curve across studies could be due to a variety of factors, including differences in strain, KO strategy, sample size, dietary maneuver including the potassium anion (21, 26), sexual dimorphism (41, 42), and circadian effects related to time of sacrifice (43), or technical details related to potassium sampling, or pNCC detection (44).

Though KS-WNK1 augments NCC phosphorylation during hypokalemia and dampens NCC phosphorylation during hyperkalemia, the mechanisms of action are distinct. During K^+^ loading, the relationship between KS-WNK1 and pNCC changes as the blood potassium concentration rises above 5.6 mmol/L. Above this breakpoint, pNCC abundance decreases dramatically, resulting in a steep negative deflection in slope. Since this feature is not present in KS-WNK1–KO mice, our findings suggest that KS-WNK1 recruits an auxiliary mechanism that promotes NCC dephosphorylation during hyperkalemia, independently of WNK4-SPAK/OSR1 signaling. The process also may be WNK body independent, as these condensates are not visible in hyperkalemic WT mice. Though the underlying mechanism is not clear, our data imply that KS-WNK1 promotes the action of NCC-specific phosphatases, especially given their established role in NCC dephosphorylation during hyperkalemia (4, 5, 45). Interestingly, Grimm et al. (45) recently identified a similar K^+^ deflection point for protein phosphatase inhibitor-1 (I-1; *PPP1R1A*), an inhibitory subunit of protein phosphatase 1A (PP1A) that is highly expressed in the DCT (5). In their study, potassium supplementation inversely correlated with I-1 protein expression and phosphorylation, thereby reducing I-1’s ability to inhibit PP1A. PP1A promotes potassium-induced dephosphorylation during hyperkalemia (5, 45). Since KS-WNK1–KO mice exhibited impaired NCC dephosphorylation above the I-1 inflection point, this suggests that KS-WNK1 may contribute to the downregulation of I-1 during hyperkalemia. The mechanism by which this might occur remains unknown and is an area of active research.

A challenge in studying KS-WNK1 in mouse kidney tissue is the inconsistent detection of KS-WNK1 protein in kidney lysates via immunoblots. This issue may arise from low protein expression, difficulties extracting KS-WNK1 from WNK bodies, and cross-reactivity of WNK1 antibodies with KS-WNK1 and L-WNK1. To address this, researchers have employed strategies to increase KS-WNK1 protein abundance while targeting bands absent in KS-WNK1–KO mice (23, 39). While these studies have identified a band, mass spectrometry has yet to confirm it as KS-WNK1. While a similar signal has been observed (albeit inconsistently) under extreme dietary K^+^ restriction, it is uniformly undetectable in WT mice on control or high K^+^ diets. Despite this, KS-WNK1 regulates NCC dephosphorylation during high K^+^ intake, suggesting that the protein is at least lowly expressed (23, 24). Like other drivers of phase transitions, perhaps KS-WNK1 binds partners within active subsaturated low abundance “clusters” (46) that somehow promote phosphatase activity under high K^+^, high chloride conditions. While the underlying mechanisms remain obscure, the relationship between KS-WNK1 and NCC dephosphorylation remains an intriguing area of research with potential insights into the role of signaling scaffolds as both activators and inhibitors of physiological processes.

Studies in K^+^-restricted KS-WNK1 5Q mutant mice demonstrate that KS-WNK1 recruits the WNK-SPAK/OSR1 pathway within WNK bodies to facilitate activation of SPAK and trafficking of SPAK to the apical membrane. Similar to other condensates that carry out normal cellular functions and enhance cellular fitness (47, 48), WNK bodies adopt a spherical morphology and exhibit reversibility, as they dissolve upon restoration of potassium (14, 23). In contrast, the 5Q puncta are nonspherical, suggesting that their material properties and deformability differ from WT WNK bodies. Furthermore, they appear to be dysfunctional, as phosphorylated SPAK is unable to leave the 5Q condensed phase and traffic to the DCT apical membrane, where it can engage with and phosphorylate NCC. Collectively these findings indicate that the 5Q mutant protein forms dysfunctional aggregates that prevent the WNK signaling pathway from activating NCC during hypokalemia. The tendency of deleterious mutations to cause condensation-prone proteins to form dysfunctional aggregates with altered in vitro morphology and material properties has been reported previously, notably with the RNA binding protein FUS (49). The KS-WNK1 5Q mutant may behave in a similar manner in vivo to adversely affect potassium homeostasis.

The molecular basis by which WNK body condensates augment WNK-SPAK/OSR1 pathway activity remains unresolved; however, our results suggests that normal KS-WNK1 exon 4a structure is required to coordinate optimal SPAK activation. Based on AlphaFold predictions (Figure 9B), we propose that the 5Q mutation disrupts an N-terminal α helix but maintains a functional C-terminal IDR that can phase separate and bind WNK body constituents, including L-WNK1, WNK4, and SPAK (12, 14). Like other poly-Q peptides (50), the stretch of consecutive N-terminal glutamines introduced by the 5Q mutation may promote the sequestration, aggregation, and diffusional arrest of KS-WNK1 binding partners, causing the protein to function like a sponge that inhibits signal transduction despite an increase in protein abundance (as suggested by increased SPAK protein abundance) (Figure 10A). Definitive testing of this hypothesis will require characterization of the material properties of WNK bodies versus 5Q assemblies, both in vivo and in vitro.

Our results also identify KS-WNK1–dependent WNK bodies as key determinants of sex-based differences in distal nephron function. Our data suggest that female mice require DCT WNK bodies to minimize salt wasting and maintain potassium homeostasis. Recent observations indicate that females prioritize distal tubule salt reabsorption via NCC more than males (41, 51). The reason for this is unclear, but a role for NCC in defending against hypokalemia during pregnancy has been proposed (52). Regardless, our data suggest that the underlying mechanism is dependent on the ability of females to leverage the distal tubule and WNK body–mediated signaling more efficiently during the threat of potassium deficiency. Given the influence on whole animal physiology, our findings more generally imply that biomolecular condensates may contribute to sexual dimorphism in other regulatory systems.

Finally, our results from normal human kidney parenchyma revealed an inverse relationship between WNK body condensate abundance and serum [K+], with WNK body abundance progressively increasing as [K+] fell below 4.0. This mimics the inverse relationship observed in mice between [K^+^] and WNK body expression. Due to a limited number of humans tissue samples, we were unable to probe for pSPAK and/or pNCC activation. Nevertheless, Thomson et al. have previously shown in human kidney tissue from patients with severe hypokalemia that there is an increase in WNK body abundance that correlates with an increase in pSPAK at the apical membrane (16). In addition, studies have demonstrated an inverse relationship between dietary potassium intake and pNCC in human urinary exosomes (53). Taken together, these studies suggest that, in humans, WNK bodies are involved in activation of the WNK-SPAK/OSR1 pathway — not just during frank hypokalemia, but also when blood potassium concentrations are in the low-to-normal reference range of 3.5–4.0 mmol/L. This raises an intriguing question about the current laboratory definition of normokalemia — is a [K^+^] of 3.5 mmol/L truly “normal” if it is sufficient to induce a stress response that promotes WNK body formation, which would predispose to BP salt sensitivity? Indeed, a recent meta-analysis comprising greater than 1 million participants found that the risk of adverse cardiovascular outcomes, end-stage kidney disease, and all-cause mortality increases as blood [K^+^] falls below 4.0 mmol/L (54). Conceivably, titrating K^+^ intake to achieve a plasma potassium concentration sufficient to inhibit WNK body condensation may be an effective natriuretic antihypertensive strategy in certain clinical scenarios (55–57).

In conclusion, our findings provide insight into the role of KS-WNK1 and WNK body condensates in potassium-dependent NCC regulation in both mice and humans. They identify KS-WNK1 as a DCT-specific amplifier that optimizes NCC reactivity to changes in systemic potassium concentrations. This observation reconciles almost two decades of conflicting data regarding stimulatory versus inhibitory effects of KS-WNK1 on NCC. Key to the amplification process is KS-WNK1’s ability to organize the WNK signaling pathway within specialized biomolecular condensates that impact potassium metabolism, BP salt sensitivity, and nephron sexual dimorphism. WNK body assembly requires KS-WNK1, which emerged during vertebrate kidney evolution from L-WNK1, a crowding-sensitive condensation-prone protein that first appeared in single-celled organisms to control salt transport and fluid volume (17, 18). Thus, it appears that through nephron segment-specific isoform expression, the mammalian kidney repurposed this ancient condensate-dependent volume regulatory system for total body potassium and BP homeostasis.

## Methods

### Sex as a biological variable.

Our study disaggregated data from male and female animals, and sexual dimorphic effects are reported.

### Mice.

KS-WNK1–KO mice and age-matched WT littermates were generated in a 129/Sv background as previously described (12, 37). For the generation of the KS-WNK1 5Q mutant mice in a 129/Sv background CRISPR/Cas9 homology-directed repair was used to knockin a mutation in exon 4a of the *WNK1* gene, replacing 5 consecutive bulky hydrophobic residues (spanning Val-11 to Val-15) with 5 neutral glutamines (Figure 9A and Supplemental Figure 8, A–D). Please see the Supplemental Methods for more detailed information.

### Dietary maneuvers.

To determine the effect of KS-WNK1 on NCC phosphorylation, mice were fed K^+^ diets for 10 days: (a) LK, (b) control K^+^, (c) HKB, (d) high KCl (Teklad). To induce hyperkalemia, mice fed the HKB diet were supplemented with amiloride (2 mg/kg/d) in their drinking water for 10 days. To measure the effect of KS-WNK1 on intake, output, and blood and urine parameters, mice were individually housed in metabolic cages (Tecniplast). After 10 days, mice were anesthetized with isoflurane, and blood was obtained via terminal cardiac puncture and analyzed by iSTAT (Abbot). Kidneys were harvested and flash frozen for immunoblot and/or paraformaldehyde-treated for microscopy. Further details can be found in the Supplemental Methods.

### Immunohistochemistry.

In accordance with the University of Pittsburgh Internal Review Board (IRB STUDY19120038), the Pitt Biospecimen Core provided formalin-fixed, paraffin-embedded nonneoplastic kidney tissue from 6 individuals who underwent radical nephrectomy for renal tumors (Figure 11). For processing and imaging information please see the Supplemental Methods.

### Immunoblotting.

For protein quantification, mouse kidney cortices were flash frozen and processed as previously described (21) and further described in the Supplemental Methods. Uniform protein loading was determined using Coomassie-stained gels, as previously described (21, 44). Next, equal amounts (20–40 μg) of protein were separated by SDS-PAGE using 4%–20% Criterion TGX precast gels (Bio-Rad). Protein was transferred to a nitrocellulose membrane. Signal densitometry was measured using Bio-Rad ChemiDoc, and densitometry was quantified with ImageLab analysis software (Bio-Rad). To plot NCC densitometry values as a function of blood [K^+^], samples from WT and mutant mice placed on control diets were run on the same gel as those from WT and mutant mice treated with a specific potassium maneuver (Supplemental Figure 2). This permitted normalization of all values to the protein abundance in WT mice on control diet. Antibodies are listed in the Supplemental Methods.

### BP telemetry.

Female KS-WNK1–KO mice and WT littermates were anesthetized with isoflurane, and DSI PA-C10 telemetry units (Data Sciences International) were surgically implanted into the femoral artery as previously described (58). BP was collected every day from 10 am to 4 pm (daytime) and 10 pm to 4 am (nighttime) for the duration of the diet challenge using Spike2 software (Cambridge Electronic Design). HCTZ challenge was performed on day 15. During the HCTZ treatment, mice were maintained on either control or LK diet with 1% saline drinking water. Daytime BP was obtained for 6 hours the day prior to HCTZ administration and on the day of HCTZ administration. Mice were injected with HCTZ (25 mg/kg i.p.) at 9 am, and then BP was collected from 10 am to 4 pm (daytime). For more details see the Supplemental Methods.

### Diuretic challenge.

Mice were placed on respective K^+^ diets for 10 days. For the first 8 days they were housed in the standard cages. On day 9, they were individually housed in metabolic cages and acclimated for 24 hours. On day 10, mice were given i.p. injections of HCTZ (25 mg/kg) and urine was collected for 6 hours. Urine [Na^+^], [K^+^], and [Cl^–^] levels were determined using Easy Lyte Plus Na/K/Cl analyzer (Medica Corp).

### Quantitative immunofluorescence confocal microscopy.

For Figure 4, WNK body analysis and quantification in formalin-fixed, paraffin-embedded mouse kidney, tissues sectioned at 5 μm thickness were processed for immunofluorescence staining according to a protocol similar to the immunohistochemistry protocol used for human tissue described above, and further details are provided in Supplemental Methods. Images were acquired with a Leica DM6000B wide-field microscope with a Retiga 4000R Fast 1394 camera. Two independent, blinded analyses were conducted to measure WNK body size and count using a custom macro in ImageJ (NIH). The results were averaged, unblinded, and plotted as a function of blood [K^+^].

For Figures 6 and 9, paraformaldehyde-fixed kidney tissues were processed and prepared as previously described (21) and further described in the Supplemental Methods. Imaging of the kidney tissue was performed using a Leica HCX PL APO CS ×40, 1.25 numerical aperture oil objective on a Leica TCS SP5 CW-STED confocal microscope utilizing Leica LAS-X software. To produce quantitative measures for pSPAK/pOSR1 puncta number, size, and distance to the DCT lumen, we used Imaris (Bitplane, v9.5) image analysis software as described in the Supplemental Methods. To measure WNK condensate morphology in 5Q mice (Figure 9), confocal images of WNK1 signal from WT and KS-WNK1 5Q mice were obtained under identical confocal settings. Images were thresholded under identical parameters to generate masks, which were then used to measure the area and roundness of individual puncta, using the Analyze Particles tool in FIJI.

### CLEM.

CLEM was performed using an innovative approach pairing high-resolution fluorescence imaging with immunogold labeling and backscattered scanning electron detection via SEM of 300 nm semithin frozen sections (Figure 4). Sections were labeled with primary antibodies against WNK1, and analysis was performed with secondary antibodies labeled with a dual 5 nm gold/Alexa Fluor 488 conjugate. Sections were scanned in toto using high-resolution fluorescence microscopy using a Nikon Ti microscope, with a ×100 1.49 objective and Photometrics 95B camera (effective pixel size = 0.07 μm). The sections were then counterstained with heavy metals (OsO_4_, Pb citrate, and Ua), critical point dried, and carbon coated prior to mounting the same coverslips imaged by light microscopy in a JEOL SEM. Following a low-magnification SEM scan using backscattered electron detection, the image was inverted and overlaid with the fluorescent image. This was used to guide nanometer resolution ultra-structural identification of WNK bodies. In these inverted electron microscopy images, dark signal corresponds to regions of high-material density. Further details are provided in the Supplemental Methods.

### Protein structure prediction.

The amino-terminal structures of WT and 5Q mutant KS-WNK1 (residues 1–72, encompassing exon 4a to the C-terminal end of the remnant kinase domain; residue 494 of Uniprot sequence Q9JIH7-1) were predicted using ColabFold (59), accessed via UCSF ChimeraX (60). Full-length L- and KS-WNK1 were rendered with AlphaFold3 (61).

### Statistics.

Data were analyzed using GraphPad Prism software and are presented as mean ± SEM, plus individual data points. Comparisons between 2 groups were determined by a Student’s 2-tailed *t* test. Comparisons between multiple groups were determined using 1- or 2-way ANOVA, followed by the appropriate post hoc test, as indicated. *P* values equal to or less than 0.05 were considered statistically significant. To analyze the relationship between blood [K^+^] and NCC, regression analyses were performed in Prism. Data were preliminarily fitted via 1-phase decay, indicating an exponential relationship with suboptimal goodness of fit. Thus, the absolute *Y* values (representing normalized protein densitometry as described above) were logarithmically transformed and analyzed by segmental versus simple linear regression. A comparison of fits was performed by *F* test, with data presented fit to the preferred regression model (alternative hypothesis = segmental vs. null hypothesis = straight line). *P* values for these comparisons are shown with the corresponding residuals in Supplemental Figure 5.

### Study approval.

For animal studies, all protocols conformed to the NIH *Guide for the Care and Use of Laboratory Animals* (National Academies Press, 2011) and were approved by the University of Pittsburgh IACUC.

For human studies, all research was approved by the University of Pittsburgh IRB, with an exemption for secondary research with data and/or specimens (IRB STUDY19120038). An IRB-approved honest broker provided the deidentified, safe harbor data, including age, sex, and serum K^+^ measured on the day of surgery.

### Data availability.

Raw and analyzed data are included in the accompanying Supporting Data Values file. Any additional information required to reanalyze the data reported in this paper is available from the corresponding author upon request.

## Author contributions

CRBS and ARS designed the study. CRBS, RTB, JAL, MNV, AB, SAK, SEG, LJN, KEQ, ALM, JF, MS, SCW, and SDS performed experiments. CRBS, DJS, MNV, NHN, AB, SDS, SAK, SCW, OBK, and ARS analyzed data. CRBS, DJS, and ARS made the figures. CRBS, RTB, SEG, DJS, SCW, ARR, SDS, OBK, and ARS drafted the paper. CLH provided KS-WNK1–KO mice. All authors approved the final version of the manuscript.

## Supplementary Material

Supplemental data

Unedited blot and gel images

Supporting data values

## Version 1 06/10/2025

In-Press Preview

## Version 2 08/01/2025

Electronic publication
