# Supplementary material for: Kidney-specific WNK1 amplifies kidney tubule responsiveness to potassium via WNK body condensates
Source: J Clin Invest. 2025 Jun 10;135(15):e188792. doi: 10.1172/JCI188792 (PMC12321387; doi:10.1172/JCI188792)
Supplement: Supplemental data [file jci-135-188792-s109.pdf]

Supplemental Data

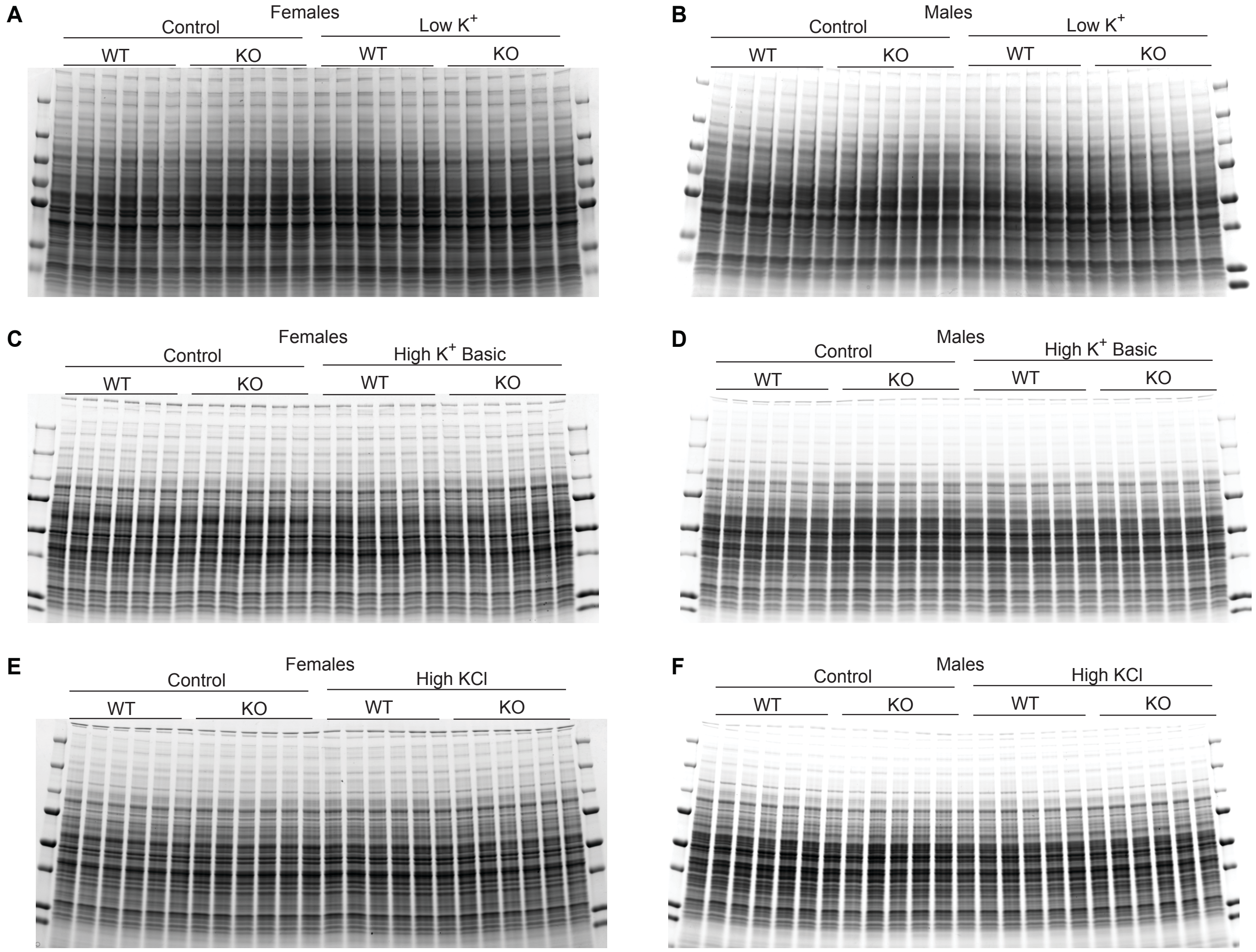

Figure S1. Optimized Coomassie gels for immunoblotting (KS-WNK1 KO experiments).
Coomassie blue stained gels, optimized to verify equal protein loading. To make comparisons between different diets, immunoblots were run against the same lysate from control WT samples in the first 12 lanes. Gels correspond to immunoblots presented in multiple figures, as noted above.
(A) Female WT and KS-WNK1 KO mice on control vs low K^+ diet.
(B) Male WT and KS-WNK1 KO mice on control vs low K^+ diet.
(C) Female WT and KS-WNK1 KO mice on control vs HKB diet.
(D) Male WT and KS-WNK1 KO mice on control vs HKB diet.
(E) Female WT and KS-WNK1 KO mice on control vs HKCl diet
(F) Male WT and KS-WNK1 KO mice on control vs HKCl diet.

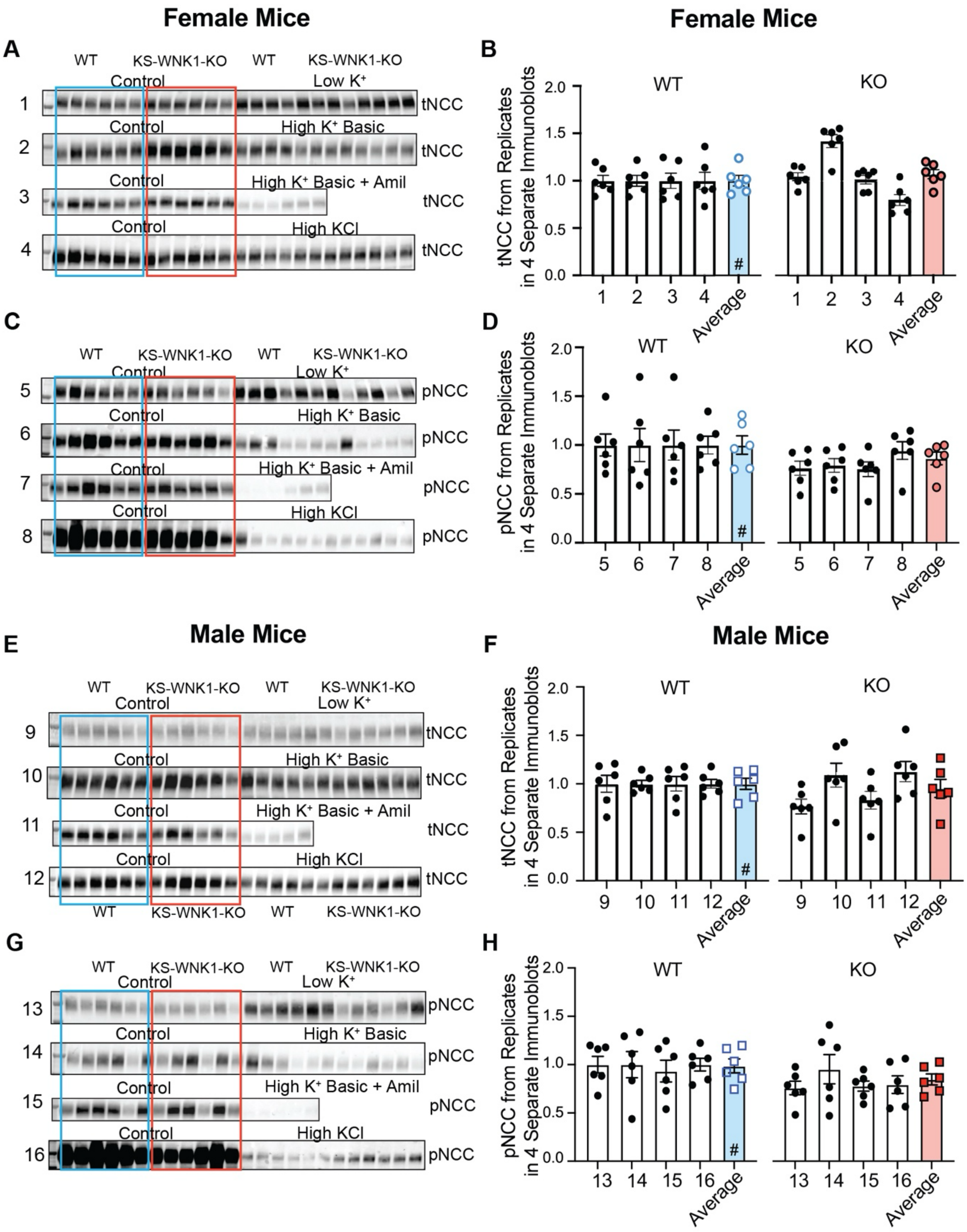

Figure S2. Normalization method for Figure 2 immunoblots using WT control lysate replicates. To analyze tNCC and pNCC densitometry values as a function of blood [K$^+$], WT and KO mice on control diets (first 12 lanes) were compared to WT and KO mice on varying K$^+$ diets (last 12 lanes). Immunoblot and corresponding graphs are shown for **A-B)** tNCC females, **C-D)** pNCC females, **E-F)** tNCC males, **G-H)** pNCC males. Each western blot for the control diet is a replicate of n = 6 WT mice and 6 KO mice (male or female). Protein abundance from varying K+ diets was normalized to WT control diet levels to calculate fold-change. Blue boxes around blots indicate WT lysate replicates. Red box indicate KO lysate replicates. #Blue bar graph represents the WT control data and red bar graph represents the KO control data shown in Fig 2. Duplicated immunoblots are from Fig 2, Fig S3, and Fig S4.

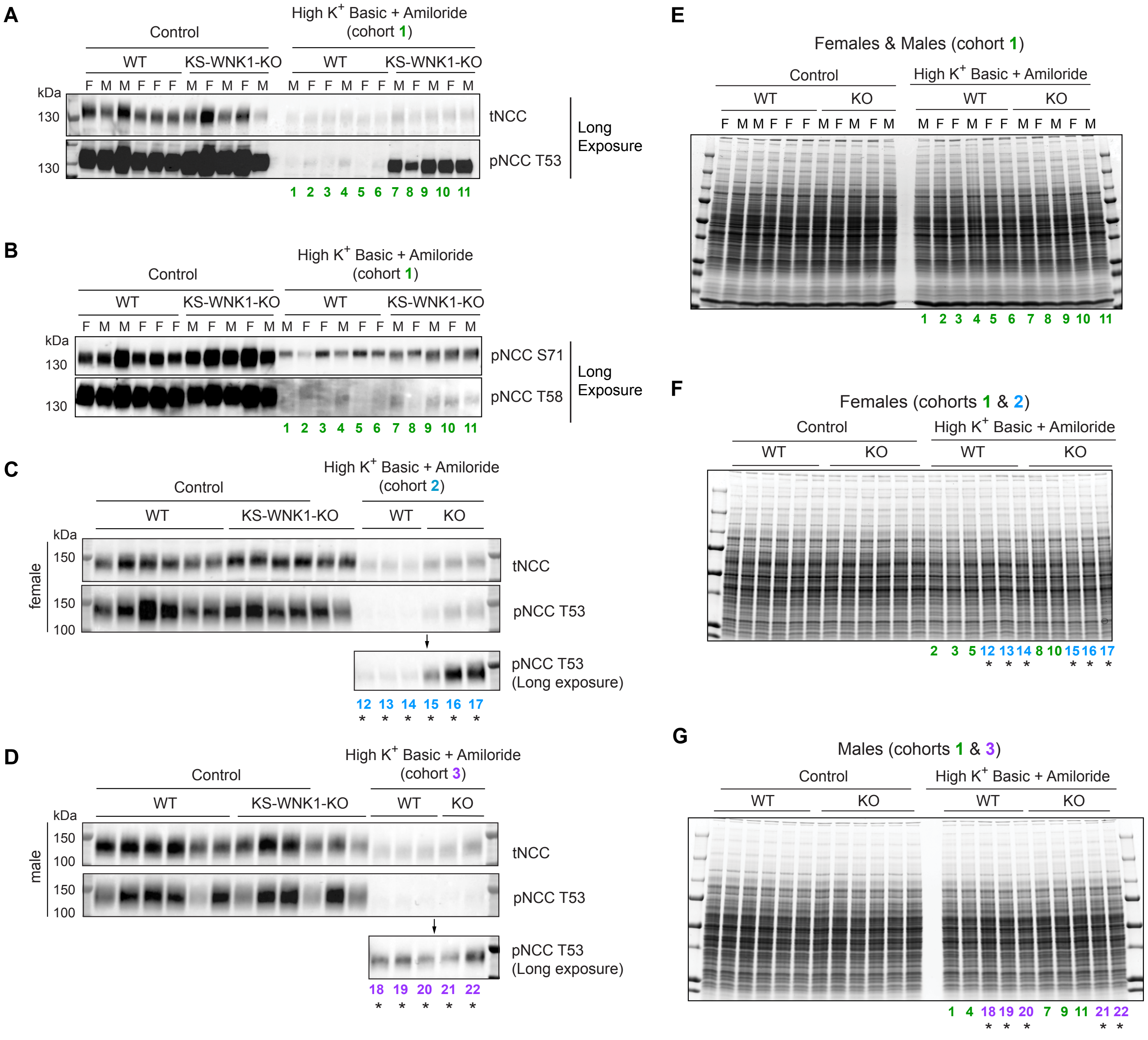

Figure S3. Effect of potassium loading plus amiloride on NCC phosphorylation in KS-WNK1 KO mice. Immunoblotting data for mice subjected to alkaline K+ loading with amiloride (2mg/kg/day) were derived from 3 separate cohorts. Cohort 1 (male + female) was studied first. Cohorts 2 (female only) and 3 (male only) were studied several months later. These additional cohorts were included to achieve sufficient n for disaggregation of results by sex. n was limited for all cohorts due to extreme hyperkalemia (Table S2). Mice from cohorts 1, 2, and 3 are indicated in green, blue, and purple, respectively. Lysates from individual mice were numbered as indicated (i.e., #1 represents the same mouse lysate in all blots). The increased signal in the KO mice treated with the HKB diet + amiloride was conspicuous after prolonged membrane exposure, as shown. **(A)** Cohort 1, with male and female protein lysates analyzed on the same blot (duplicate immunoblot from Figure 2C). Male (M) mice were normalized to male controls, and female (F) mice were normalized to female controls. Phosphorylated NCC was probed with pNCC-Thr53 antibody. **(B)** pNCC-Thr58, and pNCC-Ser71 antibodies also detected an increase in pNCC in KS-WNK1 KO mice subjected to HKB + amiloride. **(C)** Cohort 2, female mice treated with HKB + amiloride. **(D)** Cohort 3, male mice treated with HKB + amiloride. **(E-G)** Optimized Coomassie gels. Lanes indicated by asterisks (*) correspond to the mice that were used for the sex-disaggregated tNCC and pNCC immunoblots in C & D.

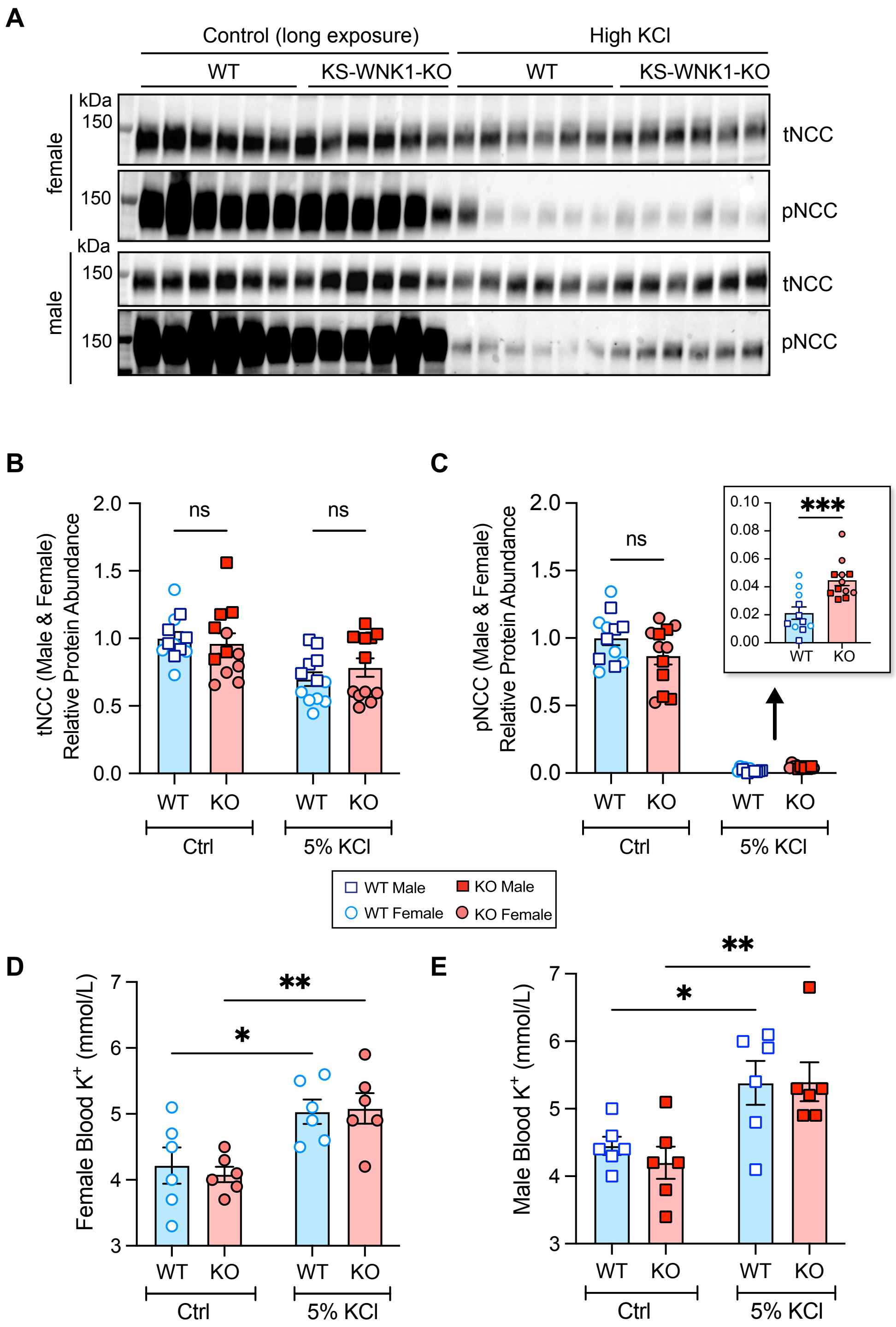

Figure S4. Effect of high KCl diet and KS-WNK1 on NCC activation.
(A) Immunoblot of kidney cortical extracts from mice treated with either control or high KCl diet for 10 days. Control diet results are shown overexposed to visualize high KCl results (duplicate immunoblot from Figure S2). **(B-C)** Quantification of immunoblots. High KCl diet significantly reduced pNCC abundance. KS-WNK1 KO mice have a blunted reduction in pNCC abundance compared to WT mice on high KCl diets. Immunoblot results are shown as means ± SE; n = 11-12 mice per genotype and diet (males and females combined). **(D-E)** Whole blood [K+] in mice treated with high KCl diets. Results are shown as means ± SE; n = 5-6 mice per genotype, sex, and diet. Two-way ANOVA with Sidak's multiple comparisons test was applied comparing WT and KS-WNK1 KO, *$P < 0.05$, **$P < 0.01$, ***$P \leq 0.001$, ****$P \leq 0.0001$.

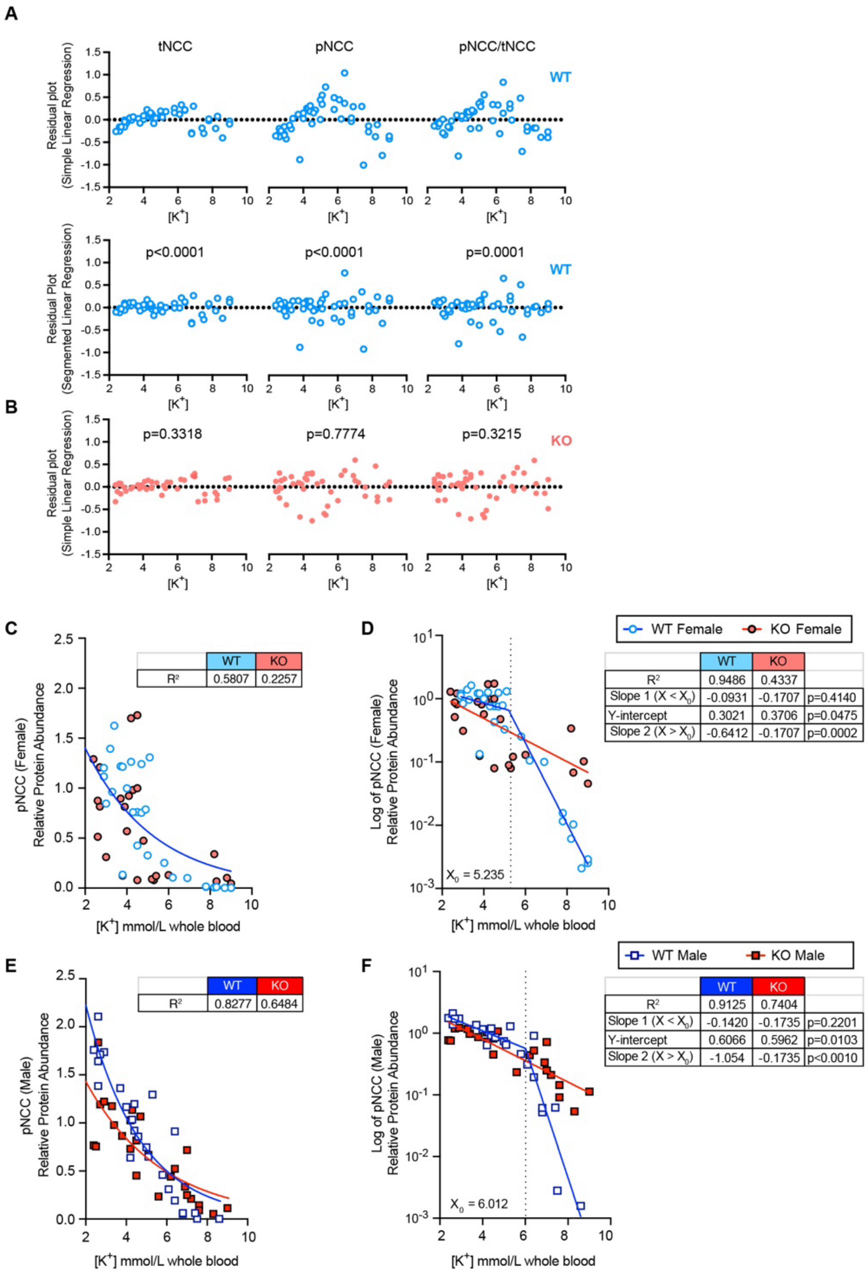

Figure S5. Residual plots for log transformed NCC data versus [K^+] in WT and KS-WNK1 KO mice.
(A) Residual plots in WT mice, modeled by simple linear regression (top) or by segmented linear regression (bottom). For the simple linear regression residuals, all three plots exhibit a nonrandom concave-down distribution relative to the horizontal dotted line, suggesting systematic inconsistencies in the models and suboptimal goodness-of-fit. When the data were re-modeled by segmental linear regression, the residuals for all three datasets were distributed normally. P-values indicate the results of a comparison-of-fits between the segmented vs simple linear models, with simple linear regression as the null hypothesis ($P \leq 0.0001$ for all three WT datasets). **(B)** Similar residual plots for KS-WNK1 KO mice, modeled by simple linear regression. In the case of KS-WNK1 KO mice, segmental linear regression did not improve the fit for KO animals and was therefore rejected, as indicated by the non-significant P-values. P-values indicate the result of a comparison-of-fits test between segmented vs simple linear regression models, with simple linear regression as the null hypothesis. **(C,E)** pNCC in female and male mice disaggregated by sex, fit to single exponential curves. For female mice one curve better fit the data, whereas for male mice two curves better fit the data. **(D, F)** Normalized pNCC densitometry in A & B were log transformed and analyzed by linear regression. WT data were best fit by a segmented linear regression regime, with X_0 breakpoints (dotted line) shown. For KO mice, the log-transformed data were best fit by simple linear regression. *P*-values represent slope comparisons between WT and KO; in the event where Slope 1 (X < X0) comparisons did not reach significance, Y-intercept comparisons with *P*-values are shown.

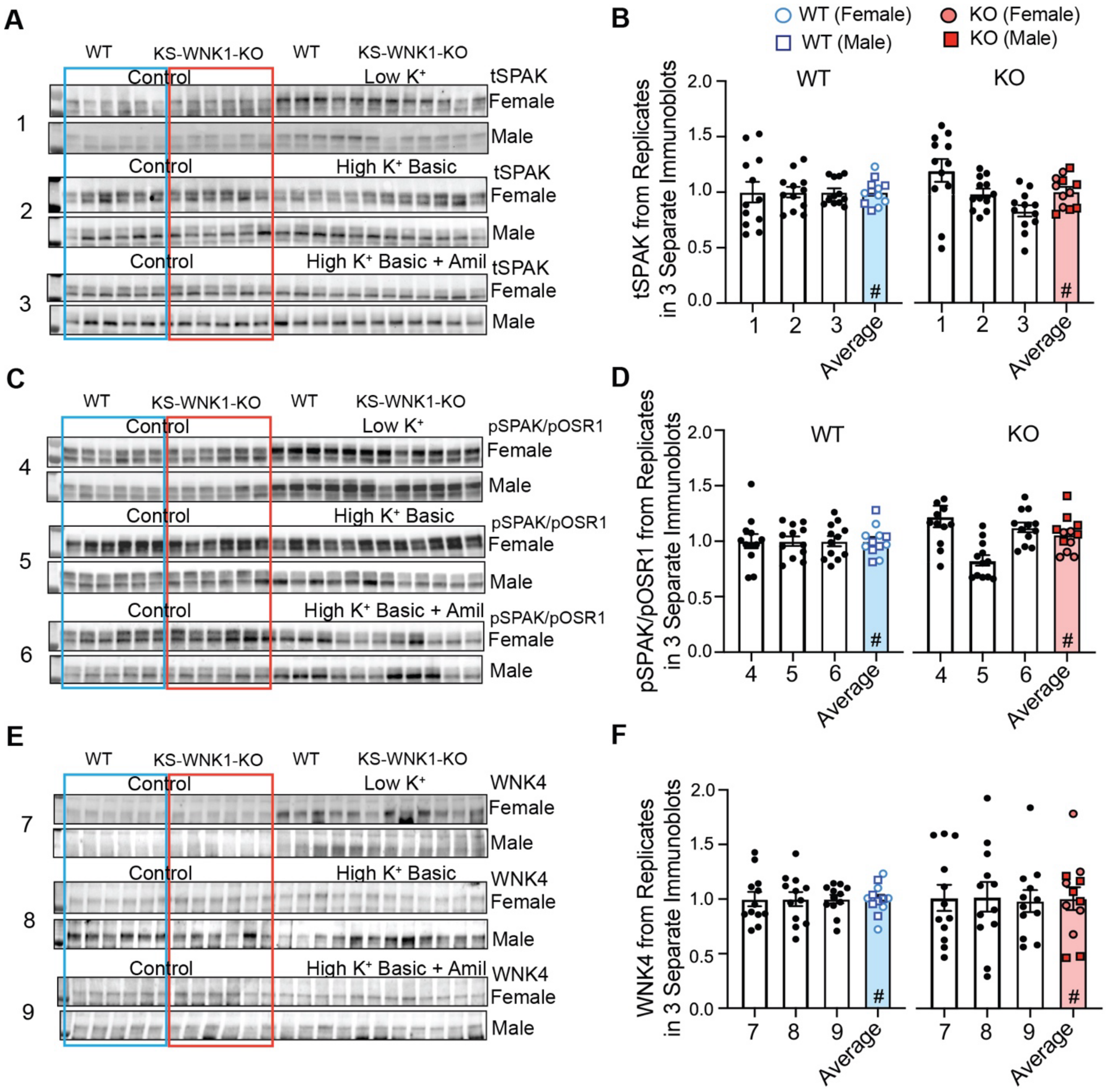

Figure S6. Normalization method for Figure 5 immunoblots using WT control lysate replicates.
To analyze SPAK, pSPAK/pOSR, and WNK4 densitometry values as a function of dietary K$^+$, WT and KO mice on control diets (first 12 lanes) were compared to WT and KO mice on varying K$^+$ diets (last 12 lanes). Immunoblot and corresponding graphs are shown for **A-B)** SPAK, **C-D)** pSPAK/pOSR1, **E-F)** WNK4. Each western blot for the control diet is a replicate of n = 12 WT mice and 12 KO mice (males and females combined). Protein abundance from varying K+ diets was normalized to WT control diet levels to calculate fold-change. Blue boxes around blots indicate WT female and male lysate replicates. Red boxes indicate KO female and male lysate replicates. #Blue bar graph represents the WT control data shown in Figure 5, and red bar graph represents the KO control data in Figure 5. Immunoblots are from Figure 5.

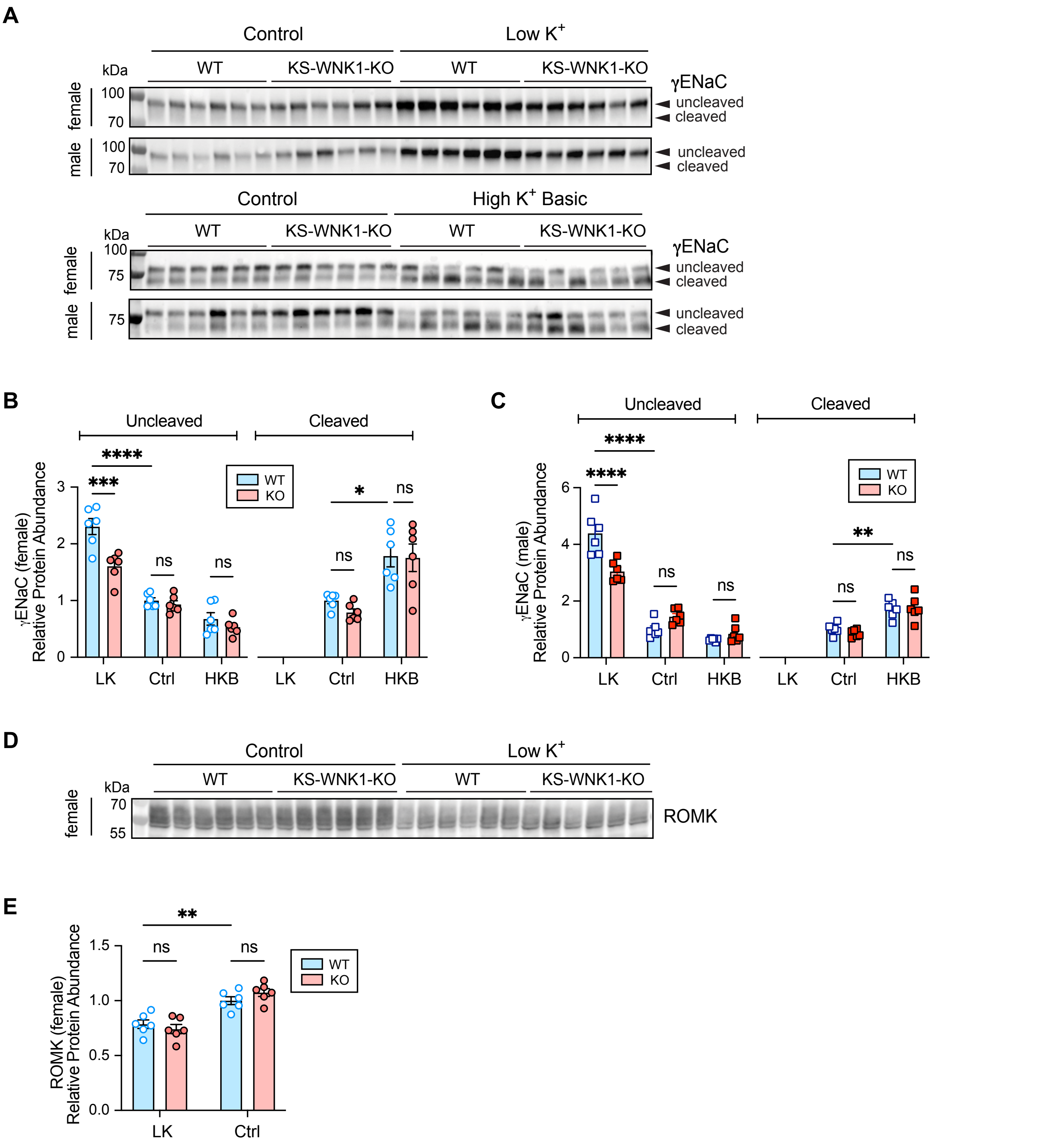

Figure S7. Effects of KS-WNK1 and dietary potassium on ENaC and ROMK protein abundance.
IB analysis of kidney cortical extracts from female and male WT and KS-WNK1 KO mice fed low K^+, control, and high K^+ basic for 10 days. **(A)** γENaC. Both the cleaved (hyperactive) and uncleaved forms are indicated with an arrow (1). **(B-C)** Bar graphs showing relative protein abundance, normalized to WT littermates on control diet. The KO mice have significantly reduced uncleaved γENaC during low K^+ diet. The cleaved form of γENaC is undetectable during low K^+ diet. **(D-E)** Low K^+ diet reduces the abundance of ROMK in female mice, however KS-WNK1 expression has no effect on ROMK protein abundance. Results are shown as mean ± SE; $n = 6$ mice per genotype, sex, and diet. Two-way ANOVA with Sidak's multiple comparisons test was applied comparing WT and KS-WNK1 KO, $*P < 0.05$, $**P < 0.01$, $***P \leq 0.001$, $****P \leq 0.0001$.

A

WT sequence (green), PAM in blue. gRNA KS-WNK1-4a-1 and KS-WNK1-4a-2

```
~~~~~~KS - Wnk1- 4a - 1~~~~>                                                          ~~~~~~KS - Wnk1- 4a - 2~~~~>
  S L L P F L M D F M K K D F C S                    V F V I V        N S H C C C C S Q               K   D
AAGTCTGTTGCCTTTTCTGA TGGATTTTATGAAGAAAGATTTTTGTT  CAGTATTTGTTATTGTA AATTCTCATTGCTGCTGCTGTTCTCAA  AAGGAT
```

WT sequence replaced with QQQQQ (red), creation of an EcoRI site (yellow highlight)

```
~~~~~~KS - Wnk1- 4a - 1~~~~>                                                          ~~~~~~KS - Wnk1- 4a - 2~~~~>
  S L L P F L M D F M K K D F C S                    Q  Q  Q  Q Q     N S H C C C C S Q               K D
AAGTCTGTTGCC ATTCCTGATGGATTTTATGAAGAAAGATTTTTGTT  CAcaacagcaacagca  gAATTCTCATTGCTG TTGCTG CTCTCAA AAGGAT
                                                                     EcoRI
```

B

PCR Primers to amplify the target region. Product length 549 bp	
Primer Pair	Sequence (5'->3')
KS-WNK1-4a-F1	CTCAGAGTTGGGAAGCATGGA
KS-WNK1-4a-R1	GCCAACTAATCTGCTGGAAACA

C

PCR genotyping (EcoRI digest)

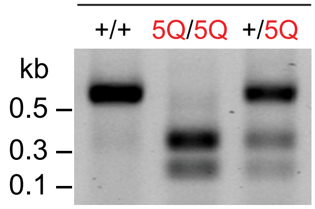

D

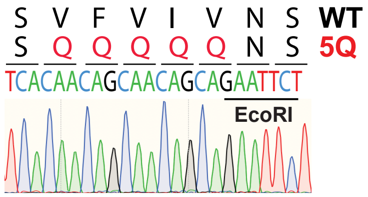

Figure S8. Generation of KS-WNK1 5Q mutant mice.
(A) The Exon 4a encoded bulky hydrophobic patch VFVIV of the CRH motif (shown in green) was replaced with QQQQQ (shown in red) via homology-directed repair (HDR). The HDR template also contained an engineered EcoRI site (yellow highlight) for genotyping purposes. Single guide RNAs flanking the hydrophobic domain are indicated as KS-WNK1-4a-1 and KS-WNK1-4a-2. The guides were injected into 129-Elite Mouse 129S2/SvPasCrl fertilized embryos, which were transferred to pseudo-pregnant female recipients. **(B)** Pups were then genotyped by PCR using the indicated primers. **(C)** PCR gel with genotyping via EcoRI digest indicating homozygous 5Q/5Q mutant mice. The founders were backcrossed with 129-Elite Mouse 129S2/SvPasCrl for 5 generations prior to experimentation. **(D)** Sanger sequencing of the TA-cloned genomic DNA PCR product confirming the correct mutation with diagnostic EcoRI site in 5Q mutant mice.

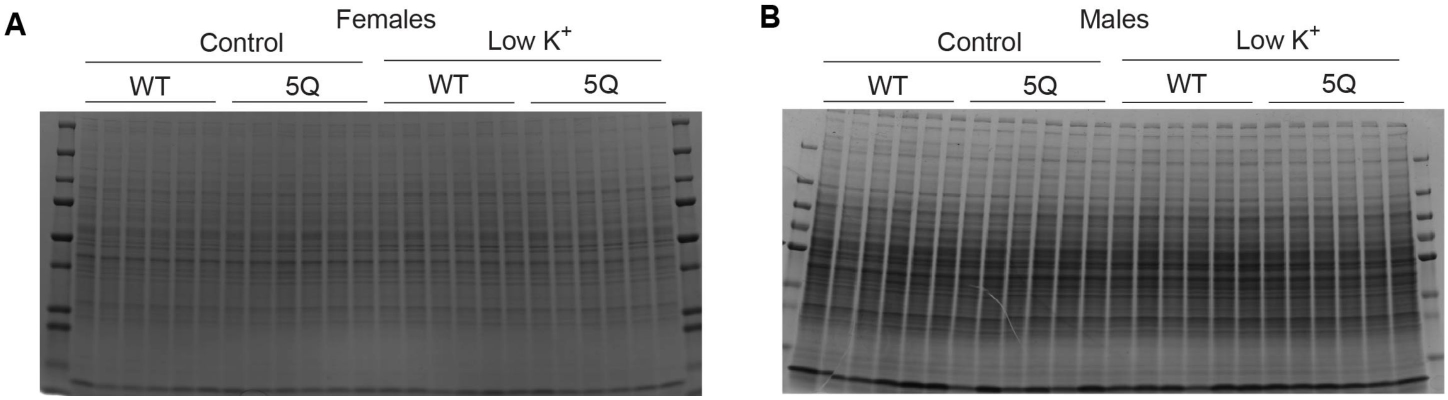

Figure S9. Potassium-restricted KS-WNK1 5Q mice: optimized Coomassie gels and data quantification disaggregated by sex.

(A-B) Coomassie blue stained gels, optimized to verify equal protein loading; **(A)** Female WT and KS-WNK1 5Q mice on control vs low K+ diet, **(B)** Male WT and KS-WNK1 5Q mice on control vs low K^+ diet. These gels correspond with Fig 10A.

Table S1: Calculated percent of minerals in the varying K^+ diets				
	Low K^+ TD.88238	Control TD.88239	High K^+ Basic TD.07278	High KCl TD.88239
Na^+	0.3%	0.3%	0.3%	0.3%
K^+	0.003%	1%	5%*	5%
Cl^-	0.45%	0.9%	2%	5.2%
Ca^{2+}	1%	1%	1%	1%
*with a 1:1:1 ratio of K^+ citrate, KCl, K^+ carbonate				

Table S1. Mineral content in the various K^+ diets used in the study.

Table S2: Effects of KS-WNK1 on whole blood parameters in mice on varying K^+ diets, disaggregated by sex									
		Low K^+		Control		High K^+ Basic		High K^+ Basic + amil	
		WT	KO	WT	KO	WT	KO	WT	KO
Female	n=	12	17	10	10	6	6	8	5
	Na^+, mmol/L	144.5 (0.3)	146.5* (0.5)	144.5 (0.7)	144.0 (0.9)	143.5 (1.1)	145.2 (1.0)	139.0 (0.3)	138.8 (1.1)
	K^+, mmol/L	3.28 (0.13)	2.91* (0.08)	4.27 (0.20)	4.19 (0.15)	5.37 (0.47)	4.67 (0.30)	8.18 (0.28)	8.06 (0.54)
	Cl^-, mmol/L	113.5 (0.7)	112.9 (0.7)	111.3 (0.6)	111.7 (0.4)	111.2 (1.1)	113.2 (1.9)	112.9 (0.8)	114.2 (1.6)
	HCO_3^-, mmol/L	22.8 (0.5)	23.2 (0.4)	23.4 (0.8)	23.2 (0.7)	22.5 (1.1)	23.5 (0.6)	22.6 (0.5)	23.2 (1.1)
	Ca^{2+}, mmol/L	1.30 (0.02)	1.28 (0.03)	1.32 (0.02)	1.32 (0.02)	1.15 (0.05)	1.22 (0.04)	1.23 (0.07)	1.27 (0.05)
	End Weight, g	26.1 (1.2)	26.1 (1.0)	26.3 (1.5)	26.1 (1.0)	25.7 (0.9)	24.8 (1.7)	20.3 (1.1)	21.5 (0.8)
	Aldo (pg/ml)	43.0 (11.5)	28.2 (14.1)	196.2 (58.8)	131.7 (37.9)				
Male	n=	10	18	12	12	6	6	5	5
	Na^+, mmol/L	144.9 (0.8)	146.8 (0.9)	143.1 (0.6)	144.1 (0.6)	144.0 (0.8)	145.3 (0.4)	139.6 (1.3)	142.4 (0.9)
	K^+, mmol/L	2.70 (0.10)	2.90 (0.09)	4.51 (0.12)	4.19 (0.12)	5.85 (0.23)	6.52* (0.23)	7.12 (0.43)	7.94* (0.32)
	Cl^-, mmol/L	109.2 (1.7)	108.0 (0.9)	112.4 (0.8)	111.8 (0.7)	110.2 (1.0)	110.8 (0.7)	107.6 (1.1)	112.0* (0.8)
	HCO_3^-, mmol/L	24.6 (1.1)	25.8 (0.5)	23.4 (0.6)	23.7 (0.3)	23.7 (0.8)	26.5* (0.4)	25.4 (0.9)	25.8 (0.8)
	Ca^{2+}, mmol/L	1.18 (0.02)	1.28* (0.02)	1.26 (0.02)	1.30 (0.02)	1.27 (0.03)	1.23 (0.01)	1.26 (0.04)	1.28 (0.02)
	End Weight, g	33.3 (1.3)	29.2 (1.0)	31.7 (1.2)	33.9 (1.4)	31.5 (1.8)	31.8 (1.4)	23.8 (1.1)	26.3 (1.6)
	Aldo (pg/ml)	13.70 (2.5)	13.9 (5.5)	153.8 (25.0)	97.4 (33.0)				
Values are means (SE): Unpaired t-test were used to determine significant differences between WT and KO on the same diet, * $P \leq 0.05$. n = as indicated, except for Aldo n= 6.									

Table S2. Effects of KS-WNK1 deletion on whole blood parameters in mice on varying K^+ diets.

Table S3: Effects of KS-WNK1 on urine parameters in mice on varying K^+ diets, disaggregated by sex		Low K^+		Control		High K^+ Basic	
		WT	KO	WT	KO	WT	KO
Female	n=	9	9	5	6	4	5
	Water intake, ml/d	4.6 (0.8)	6.5 (0.7)	3.1 (0.4)	3.1 (0.6)	6.6 (1.7)	6.0 (0.5)
	Urine Vol, ml/d	3.3 (0.5)	3.2 (0.4)	2.5 (0.5)	2.5 (0.6)	4.3 (0.8)	3.3 (0.3)
	UNaV, mmol/d	0.43 (0.09)	0.31 (0.02)	0.44 (0.07)	0.40 (0.06)	0.28 (0.03)	0.26 (0.03)
	UKV, mmol/d	0.016 (0.00)	0.015 (0.00)	0.45 (0.08)	0.41 (0.07)	1.84 (0.27)	1.96 (0.20)
	UClV, mmol/d	0.48 (0.07)	0.51 (0.05)	0.72 (0.10)	0.58 (0.19)	1.09 (0.13)	1.12 (0.11)
	U Osms,mOsms/kg	1086 (47)	922* (41)	1480 (53)	1437 (81)*	1374 (180)	1944* (121)
	Spot Urine pH	6.7 (0.1)	7.0 (0.2)	6.0 (0.1)	5.8 (0.1)	8.3 (0.3)	7.8 (0.4)
Male	n=	6	9	6	6	6	6
	Water intake, ml/d	7.2 (0.5)	7.0 (0.7)	3.9 (0.7)	3.6 (0.4)	13.0 (0.6)	13.3 (0.9)
	Urine Volume, ml/d	4.5 (0.6)	4.6 (0.4)	3.3 (0.2)	3.6 (0.5)	6.5 (0.1)	6.2 (0.4)
	UNaV, mmol/d	0.73 (0.06)	0.63 (0.09)	0.42 (0.05)	0.34 (0.04)	0.40 (0.04)	0.32 (0.04)
	UKV, mmol/d	0.003 (0.00)	0.012 (0.00)	0.90 (0.08)	0.81 (0.08)	3.23 (0.17)	2.99 (0.21)
	UClV, mmol/d	0.63 (0.06)	0.50 (0.03)	0.67 (0.18)	0.71 (0.09)	1.73 (0.09)	1.49 (0.10)
	U Osms, mOsms/kg	831 (56)	725 (42)	1221 (29)	1268 (45)	1392 (62)	1250 (36)
	Spot Urine pH	7.3 (0.1)	7.2 (0.2)	5.7 (0.1)	5.8 (0.1)	6.2 (0.1)	7.3* (0.2)
Values are means (SE): Unpaired t-test were used to determine significant differences between WT and KO on the same diet, * P ≤ 0.05.							

Table S3. Effects of KS-WNK1 deletion on urine parameters in mice on varying K^+ diets

Table S4: Effects of 5Q on whole blood parameters in mice on varying K^+ diets		Low K^+		Control	
		WT	5Q	WT	5Q
Female	n=	8	8	6	6
	Na^+, mmol/L	143.4 (0.7)	144.1 (1.1)	144.3 (0.4)	143.5 (0.7)
	K^+, mmol/L	3.3 (0.18)	2.80* (0.15)	4.63 (0.20)	4.02 (0.31)
	Cl^-, mmol/L	115.0 (1.4)	109.5** (1.2)	114.8 (1.1)	116.3 (1.2)
	HCO_3^-, mmol/L	20.1 (1.0)	24.6** (1.0)	22.0 (1.2)	20.2 (0.9)
	Ca^{2+}, mmol/L	1.17 (0.05)	1.21** (0.04)	1.31 (0.03)	1.21 (0.08)
	End Weight, g	27.0 (1.5)	24.5** (1.1)	28.3 (0.7)	26.8 (1.8)**
Male	n=	7	7	6	6
	Na^+, mmol/L	147.6 (0.8)	146.3 (0.5)	146.8 (1.2)	147.0 (1.1)
	K^+, mmol/L	2.97 (0.11)	2.80 (0.16)	4.80 (0.11)	4.75 (0.29)
	Cl^-, mmol/L	108.3 (1.0)	107.3 (2.0)	111.8 (0.8)	115.7** (0.8)
	HCO_3^-, mmol/L	25.1 (0.8)	25.0 (1.3)	26.5 (1.1)	23.0* (0.9)
	Ca^{2+}, mmol/L	1.25 (0.02)	1.21 (0.01)	1.31 (0.04)	1.28 (0.03)
	End Weight, g	28.1 (2.1)	26.4 (1.5)	28.3 (2.6)	29.2 (1.7)
Values are means (SE): Unpaired t-test were used to determine significant differences between WT and 5Q on the same diet.					

Table S4. Effects of the KS-WNK1 5Q mutation on whole blood parameters in mice on varying K^+ diets.

Supplemental Methods

Mice

KS-WNK1 knockout mice (KS-WNK1 KO) and age-matched wild-type littermates (WT) were generated in a 129/Sv background (Charles River, Wilmington, MA) as previously described (2, 3). KS-WNK1 KO mice were derived from a mouse line originally reported by Liu *et al.* (2) and described in the supplemental materials from Boyd-Shiwarski *et al.* (3). Genotyping was performed as reported by these studies previously. CRISPR-Cas9 homology-directed repair was used to knock-in a mutation in exon 4a of the *WNK1* gene, replacing five consecutive bulky hydrophobic residues (spanning Val-11 to Val-15) with five neutral glutamines, resulting in the generation of KS-WNK1 "5Q" mutant mice (Figure 9A, S8, A-D). The 5Q mutant mice were generated and bred in a 129/Sv background. Experiments were performed on both female (20-30g) and male mice (25-35g), aged 10-25 weeks. All mice were housed in a temperature-controlled room on a 12h light/dark cycle. Mice had free access to deionized water, unless otherwise noted.

Dietary Maneuvers

To determine the effect of KS-WNK1 on NCC phosphorylation, mice were fed K^+ diets for 10 days: (1) low K^+ (LK, TD.88239), (2) control K^+ (ctrl, TD.88238), (3) high K^+ basic, (HKB, TD.07278), (4) high KCl, (HKCl, TD.09075) (Teklad, Madison, WI). See Table S1 for Teklad Diet concentrations of the following minerals: Na^+, K^+, Cl^-, Ca^{2+}. To induce hyperkalemia, mice fed the high K^+ basic diet were supplemented with amiloride (2mg/kg/d) in their drinking water for 10 days. After 10 days, mice were anesthetized with isoflurane, and blood was obtained via terminal cardiac puncture and analyzed by iSTAT (Abbot). All mice had access to food and water until time of sacrifice and sacrificed the same time of day (between 1-3pm). Kidneys were harvested and flash frozen for immunoblot and/or paraformaldehyde-treated for microscopy. Urine was

immediately collected from mouse bladder for urine pH measurements using AimStrip US-5 (Germaine Inc, San Antonio, TX). Blood plasma was isolated by centrifugation and aldosterone levels were measured using ELISA kit (ENZO, ADI-900-173; sensitivity= 4.7pg/mL; intra- and inter-assay coefficient of variance <6.6% and <18%, respectively).

Metabolic Cages

To measure the effect of KS-WNK1 on intake, output, and blood and urine parameters, mice were individually housed in metabolic cages (Tecniplast, Italy). Mice were fed pellet-based diets during days 1-7, introduced to powder diets on day 7, and then switched to exclusive powder-diets for days 8-10. Powder diets were derived from blended commercial diets combined with water that was allowed to evaporate to form a solid that was simple to weigh and use in the metabolic cage. The mice were acclimated in metabolic cages for the first 24h (day 9), followed by 24h measurement of food and water intake and urine collection (day 10). Urine [Na^+], [K^+], and [Cl^-] were determined using Easy Lyte Plus Na/K/Cl analyzer (Medica Corp, Bedford, MA). Urine osmolality was determined using a micro osmometer (Precision Systems, Natick, MA). After 10 days mice were anesthetized with isoflurane, and blood was obtained via terminal cardiac puncture and analyzed by iSTAT (Abbot). Kidneys were processed for immunoblot and imaging as stated below.

Immunohistochemistry

The Pitt Biospecimen Core provided formalin-fixed, paraffin-embedded non-neoplastic kidney tissue from 6 subjects who underwent radical nephrectomy for renal tumors (Figure 11). Formalin-fixed, paraffin-embedded human kidney tissue sectioned at 5-µm thickness was processed for immunohistochemistry according to our previously published methods (3). Briefly, sections were deparaffinized and hydrated for retrieval of antigenic sites before inactivation of endogenous

peroxidases. After blocking, slides were incubated in primary antibody against WNK1 or NCC (see antibodies section) overnight, and subsequent incubation with biotinylated secondary antibodies (Jackson Immunoresearch) and ABC reagent were used to visualize staining with diaminobenzadine (Vector Laboratories). Images were acquired with a Leica DM6000B widefield microscope with a Retiga 4000R Fast 1394 camera. Images were obtained with Volocity 6.3, and analysis was performed using ImageJ/Fiji (NIH).

Immunoblotting

For protein quantification, kidney cortexes were flash frozen and processed as previously described (4). Ice-cold RIPA buffer (Thermo Scientific) was used for protein extraction with freshly added protease and phosphatase inhibitor cocktail. Protein quantification was determined using the Pierce BCA Protein Assay Kit (Thermo Scientific). Uniform protein loading was determined using Coomassie-stained gels, as previously described (4, 5). 15µg protein from each sample was loaded onto the SDS-PAGE gel and then stained with Coomassie blue. Five random bands were quantified to determine uniform loading, defined as signal variability per lane of less than or equal to 5%. If necessary, protein assays were repeated, and appropriate adjustments were made until loading across samples was determined to be uniform. Final Coomassie optimized gels are shown in Figures S1, A-F, S3, E-G, S9, A-B. Next, equal amounts (20-40µg) of protein were separated by SDS-PAGE using 4-20% Criterion TGX precast gels (Bio-Rad). Protein was transferred to a nitrocellulose membrane. Signal densitometry was measured using Bio-Rad ChemiDoc and densitometry was quantified with ImageLab analysis software (Bio-Rad). Two different protein ladders were used: Precision Plus All Blue (Biorad) and PageRuler Plus (Thermo Scientific).

To plot NCC densitometry values as a function of blood [K^+], WT and mutant mice placed on control diets were run on the same gel as WT and mutant mice treated with a specific potassium maneuver (Figure S2). This permitted normalization of all values to the protein abundance in WT mice on control diet. All normalized densitometry values were disaggregated by sex: males subjected to potassium maneuvers were normalized to WT control diet-treated males, and females subjected to potassium maneuvers were normalized to WT control diet-treated females. The densitometry values for each band – derived from the kidney lysate from one mouse – was cross referenced with the blood potassium concentration measured in that mouse at the time of sacrifice. The normalized densitometry values were then plotted with blood [K^+] on the X axis and protein abundance on the Y axis.

Antibodies

The following antibodies were used for immunoblot and/or immunofluorescence: Sodium chloride cotransporter (NCC; provided by David Ellison(6)); Sodium chloride cotransporter (NCC; Millipore Ab3553 (7)); Phospho-NCC Thr 53 (pNCC; Phospho-solutions P1311-53 (8)); Phospho-NCC Ser 71 and Phospho-NCC Thr 58 (pNCCSer71 & pNCCThr58, provided by Jan Loffing (9)); SPAK (SPAK; Cell Signaling (7)); Phospho-Ser 373 SPAK/ Phospho-Ser 325 OSR1 (pSPAK/pOSR1; Millipore 07-2273 (10)); With-no-lysine kinase 1 (WNK1; Atlas Antibodies HPA059157 (3)); With-no-lysine kinase 4, (WNK4 (3)); Gamma subunit epithelial sodium channel (γENaC; 83 kDa cleaved and 95 kDa uncleaved; Stressmarq SPC-405 (4)); ATP-sensitive inward rectifier potassium channel 10 (Kir4.1; Alomone APC-035 (11)); Renal outer medullary potassium channel (ROMK; 50-65 kDa complex glycosylated; R-80 was provided by James Wade (12)).

Blood Pressure Telemetry

Female KS-WNK1 KO mice and WT littermates were anesthetized with isoflurane and DSI PA-C10 telemetry units (Data Sciences International, New Brighton, MN, USA) were surgically implanted into the femoral artery as previously described (13). Mice then recovered for 1 week before dietary challenges were commenced. Blood pressure was collected every day from 10am-4pm (daytime) and 10pm-4am (nighttime) for the duration of the diet challenge using Spike2 software (Cambridge Electronic Design). Mean arterial pressure (MAP) was calculated by diastolic plus one-third of the pulse pressure. For the saline challenge, mice were maintained on varying K+ diets for 10 days and were then challenged with 1% saline in their drinking water for 72h. HCTZ-challenge was performed on day 15. During the HCTZ treatment mice were maintained on either control or low K+ diet with 1% saline drinking water. Daytime blood pressure was obtained for 6h the day prior to HCTZ administration and on the day of HCTZ administration. Mice were injected with hydrochlorothiazide (25mg/kg IP) at 9am and then blood pressure was collected from 10am-4pm (daytime).

Quantitative Immunofluorescence Confocal Microscopy

Paraformaldehyde fixed kidney tissues were processed and prepared as previously described (4). 6μm sections were rehydrated and treated with 1% SDS for 10 min for retrieval of antigenic sites. Then slides were washed with high salt buffer + bovine serum albumin before the addition of primary antibody. Primary antibodies were incubated overnight at 4°C, followed by washes with high salt buffer + bovine serum albumin and subsequent incubation with secondary antibodies and TO-PRO-3 Iodide for visualize staining. Imaging of the kidney tissue was performed using a Leica HCX PL APO CS x40, 1.25 numerical aperture oil objective on a Leica TCS SP5 CW-STED confocal microscope utilizing Leica LAS-X software.

To produce quantitative measures for pSPAK/pOSR1 puncta number, size, and distance to the DCT lumen we used Imaris (Bitplane, v9.5) image analysis software. Fluorescence images were first imported into Imaris. The pSPAK/pOSR1 puncta were detected using the Spots element creation wizard. A region of interest was specified to isolate a single DCT within an image. The pSPAK/pOSR1 channel was then selected as the fluorescence reference channel for both Spot identification and to guide the puncta diameter determination. A local contrast fluorescence intensity threshold was chosen, and the Spots elements were filtered using the Quality metric intrinsic to Imaris. The region growing method was used to obtain Spots of varying size. Once defined, the same quality metric and threshold settings were used throughout all puncta analysis. To not exclude data, the quality and thresholding was set to bias towards over detection. Manual editing of the Spot objects was performed to eliminate rare errant Spots when necessary. For each DCT the number of cell nuclei were counted and used to calculate the average number of pSPAK/pOSR1 puncta per cell. To measure the distance between the identified spots and the DCT lumen, we created a Surface object of the lumen. Using the Surface wizard, a Surface object was defined using the magic wand and isolines functions within the manual surface creation. This was able to reliably detect the lack of fluorescence intensity within the DCT lumen. A built-in Imaris Xtension was implemented to calculate the shortest distance between all Spots objects and the DCT lumen Surface. These data were then displayed in Imaris as Spot objects with color coded statistic values for puncta diameter or distance to DCT lumen. To measure WNK condensate morphology in 5Q mice, confocal images of WNK1 signal from WT and KS-WNK1 5Q mice were obtained under identical confocal settings. Images were thresholded under identical parameters to generate masks (Figure 9C), which were then used to measure the area and roundness of individual puncta, using the “Analyze Particles” tool in FIJI.

For WNK body analysis and quantification in formalin-fixed, paraffin-embedded mouse kidney, tissues sectioned at 5-µm thickness were processed for immunofluorescence staining according to a protocol similar to the immunohistochemistry protocol described above. After a 1-hour blocking step with donkey serum, slides were incubated in primary antibodies against WNK4 or NCC (see antibodies section) overnight, and a subsequent 2-hour incubation with fluorescent-tagged secondary antibodies (Jackson Immunoresearch) was used to visualize staining. Images were acquired with a Leica DM6000B widefield microscope with a Retiga 4000R Fast 1394 camera. Up to ten images of representative distal convoluted tubules (DCTs) per animal were captured using ImageJ. Two independent, blinded analyses were conducted to measure WNK body size and count using a custom macro in ImageJ. Threshold values were adjusted to exclude background staining, with the same settings applied consistently across tubules from the same animal. The results from both analyses were averaged, unblinded, and plotted as a function of blood [K^+].

CLEM

Correlative Light and Electron Microscopy (CLEM) was performed using a novel approach pairing high resolution fluorescence imaging with immunogold labelling and backscattered scanning electron (BSE) detection via scanning electron microscopy (SEM) of 300nm semithin frozen sections. Murine kidneys were fixed by perfusion with 2% paraformaldehyde (supplier) containing 0.1% glutaraldehyde (supplier), diced into small (2mm) cubes, cryoprotected in 2.3M sucrose, mounted on cryostubs and shock frozen in liquid nitrogen. Semithin (300nm) sections were cut using a ultracryomicrotome (Leica Ultracut 7) and mounted on #1.5 coverslips. Sections were labelled with primary antibodies to WNK1 revealed with secondary antibodies labelled with a dual 5nm gold/alexa 488 conjugate as well as cy3 Phalloidin and Hoechst dye to highlight cellular structures. Sections were scanned *in toto* using high resolution fluorescence microscopy using a Nikon Ti microscope, with a 100x 1.49 objective and Photometrics 95B camera (effective pixel

size =0.07um). The sections were then counterstained with heavy metals (OsO_4, Pb citrate and Ua) critical point dried and carbon coated prior to mounting the same coverslips imaged by light microscopy in a JEOL SEM. Following a low magnification SEM scan using backscattered electron detection, the image was inverted and overlaid with the fluorescent image. This was used to guide nanometer resolution ultra structural identification of WNK bodies. In these inverted EM images, dark signal corresponds to regions of high material density.

Protein structure prediction

The amino-terminal structures of WT and 5Q mutant KS-WNK1 (residues 1-72, encompassing exon 4a to the C-terminal end of the remnant kinase domain; residue 494 of Uniprot sequence Q9JIH7-1) were predicted using ColabFold (14), accessed via UCSF ChimeraX (15). Full-length L- and KS-WNK1 were rendered with AlphaFold3 (16).

Supplemental Reference

1. Bruns JB, Carattino MD, Sheng S, Maarouf AB, Weisz OA, Pilewski JM, et al. Epithelial Na+ channels are fully activated by furin- and prostasin-dependent release of an inhibitory peptide from the gamma-subunit. *J Biol Chem.* 2007;282(9):6153-60.
2. Liu Z, Xie J, Wu T, Truong T, Auchus RJ, and Huang CL. Downregulation of NCC and NKCC2 cotransporters by kidney-specific WNK1 revealed by gene disruption and transgenic mouse models. *Hum Mol Genet.* 2011;20(5):855-66.
3. Boyd-Shiwarski CR, Shiwarski DJ, Roy A, Namboodiri HN, Nkashama LJ, Xie J, et al. Potassium-regulated distal tubule WNK bodies are kidney-specific WNK1 dependent. *Mol Biol Cell.* 2018;29(4):499-509.
4. Boyd-Shiwarski CR, Weaver CJ, Beacham RT, Shiwarski DJ, Connolly KA, Nkashama LJ, et al. Effects of extreme potassium stress on blood pressure and renal tubular sodium transport. *Am J Physiol Renal Physiol.* 2020;318(6):F1341-F56.
5. McDonough AA, Veiras LC, Minas JN, and Ralph DL. Considerations when quantitating protein abundance by immunoblot. *Am J Physiol Cell Physiol.* 2015;308(6):C426-33.
6. Bostanjoglo M, Reeves WB, Reilly RF, Velazquez H, Robertson N, Litwack G, et al. 11Beta-hydroxysteroid dehydrogenase, mineralocorticoid receptor, and thiazide-sensitive Na-Cl cotransporter expression by distal tubules. *Journal of the American Society of Nephrology : JASN.* 1998;9(8):1347-58.
7. Chen JC, Lo YF, Lin YW, Lin SH, Huang CL, and Cheng CJ. WNK4 kinase is a physiological intracellular chloride sensor. *Proc Natl Acad Sci U S A.* 2019.

8. McCormick JA, Nelson JH, Yang CL, Curry JN, and Ellison DH. Overexpression of the sodium chloride cotransporter is not sufficient to cause familial hyperkalemic hypertension. *Hypertension (Dallas, Tex : 1979).* 2011;58(5):888-94.
9. Sorensen MV, Grossmann S, Roesinger M, Gresko N, Todkar AP, Barmettler G, et al. Rapid dephosphorylation of the renal sodium chloride cotransporter in response to oral potassium intake in mice. *Kidney Int.* 2013;83(5):811-24.
10. Thomson MN, Cuevas CA, Bewarder TM, Dittmayer C, Miller LN, Si J, et al. WNK bodies cluster WNK4 and SPAK/OSR1 to promote NCC activation in hypokalemia. *Am J Physiol Renal Physiol.* 2020;318(1):F216-F28.
11. Cuevas CA, Su XT, Wang MX, Terker AS, Lin DH, McCormick JA, et al. Potassium Sensing by Renal Distal Tubules Requires Kir4.1. *Journal of the American Society of Nephrology : JASN.* 2017;28(6):1814-25.
12. Wade JB, Fang L, Coleman RA, Liu J, Grimm PR, Wang T, et al. Differential regulation of ROMK (Kir1.1) in distal nephron segments by dietary potassium. *Am J Physiol Renal Physiol.* 2011;300(6):F1385-93.
13. Ong J, Kinsman BJ, Sved AF, Rush BM, Tan RJ, Carattino MD, et al. Renal sensory nerves increase sympathetic nerve activity and blood pressure in 2-kidney 1-clip hypertensive mice. *J Neurophysiol.* 2019;122(1):358-67.
14. Mirdita M, Schutze K, Moriwaki Y, Heo L, Ovchinnikov S, and Steinegger M. ColabFold: making protein folding accessible to all. *Nat Methods.* 2022;19(6):679-82.
15. Meng EC, Goddard TD, Pettersen EF, Couch GS, Pearson ZJ, Morris JH, et al. UCSF ChimeraX: Tools for structure building and analysis. *Protein Sci.* 2023;32(11):e4792.
16. Abramson J, Adler J, Dunger J, Evans R, Green T, Pritzel A, et al. Accurate structure prediction of biomolecular interactions with AlphaFold 3. *Nature.* 2024;630(8016):493-500.
