# Supplementary material for: Kidney-specific WNK1 amplifies kidney tubule responsiveness to potassium via WNK body condensates
Source: J Clin Invest. 2025 Jun 10;135(15):e188792. doi: 10.1172/JCI188792 (PMC12321387; doi:10.1172/JCI188792)

Figure 2 Uncropped Blots

2A

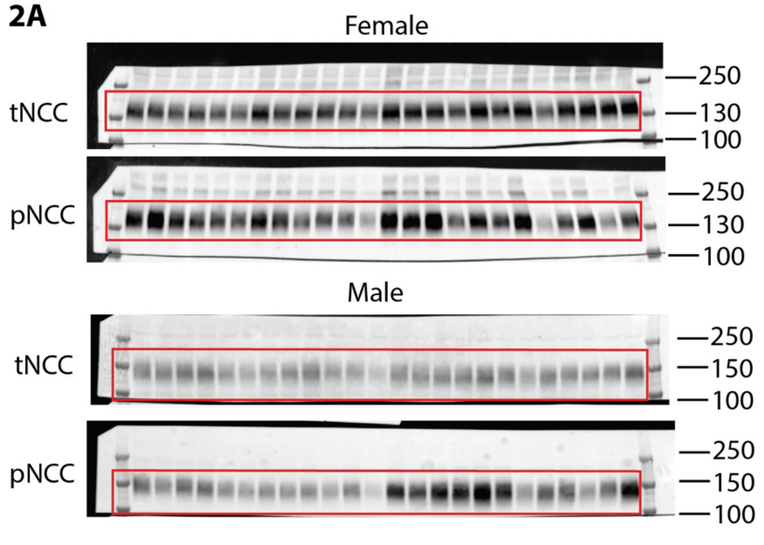

Fig 2A tNCC is duplicated in Fig S2A blot #1

Fig 2A pNCC is duplicated in Fig S2C blot #5

Fig 2A tNCC is duplicated in Fig S2E blot #9

Fig 2A pNCC is duplicated in Fig S2G blot #13

2B

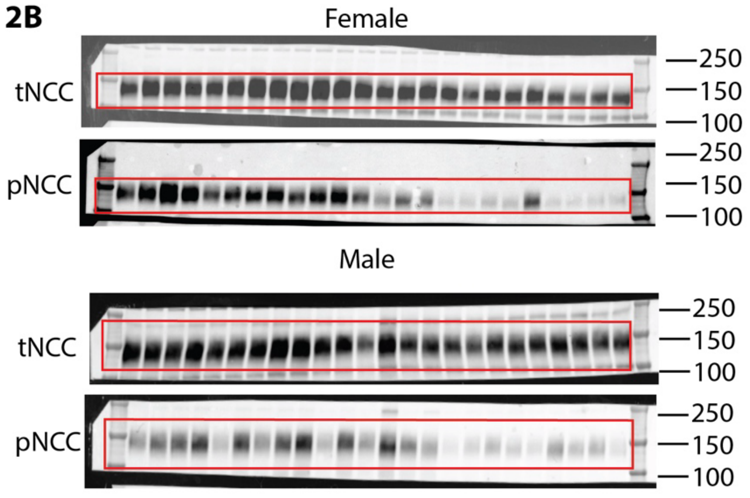

Fig 2B tNCC is duplicated in Fig S2A blot #2

Fig 2B pNCC is duplicated in Fig S2C blot #6

Fig 2B tNCC is duplicated in Fig S2E blot #10

Fig 2B pNCC is duplicated in Fig S2G blot #14

2C

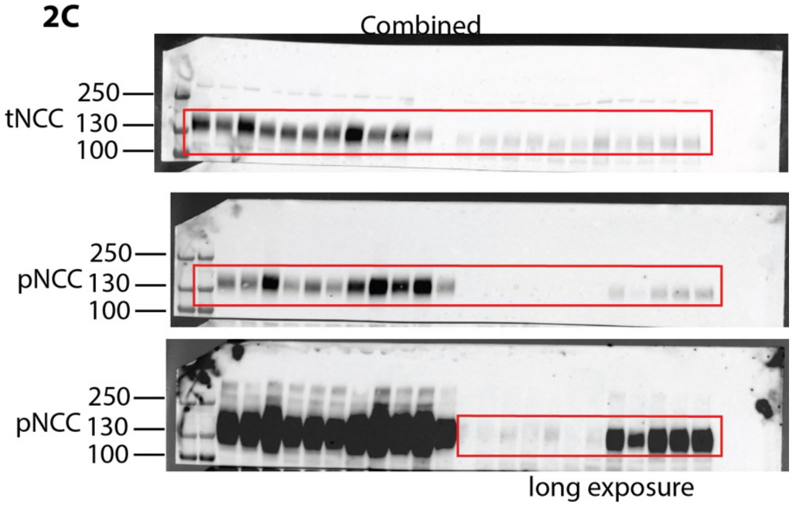

Fig 2C is duplicated in Fig S3A

Figure 5A Uncropped Blots

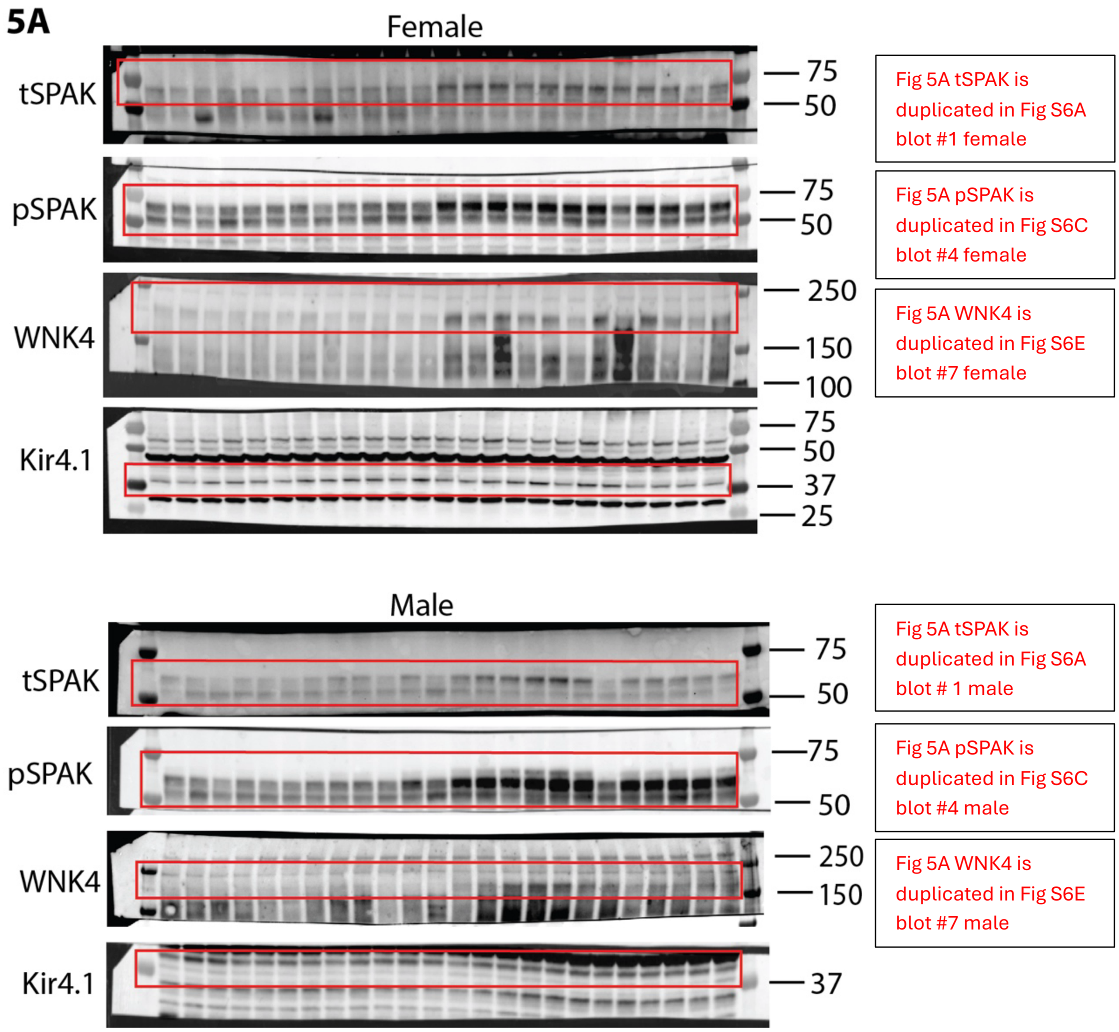

Figure 5B Uncropped Blots

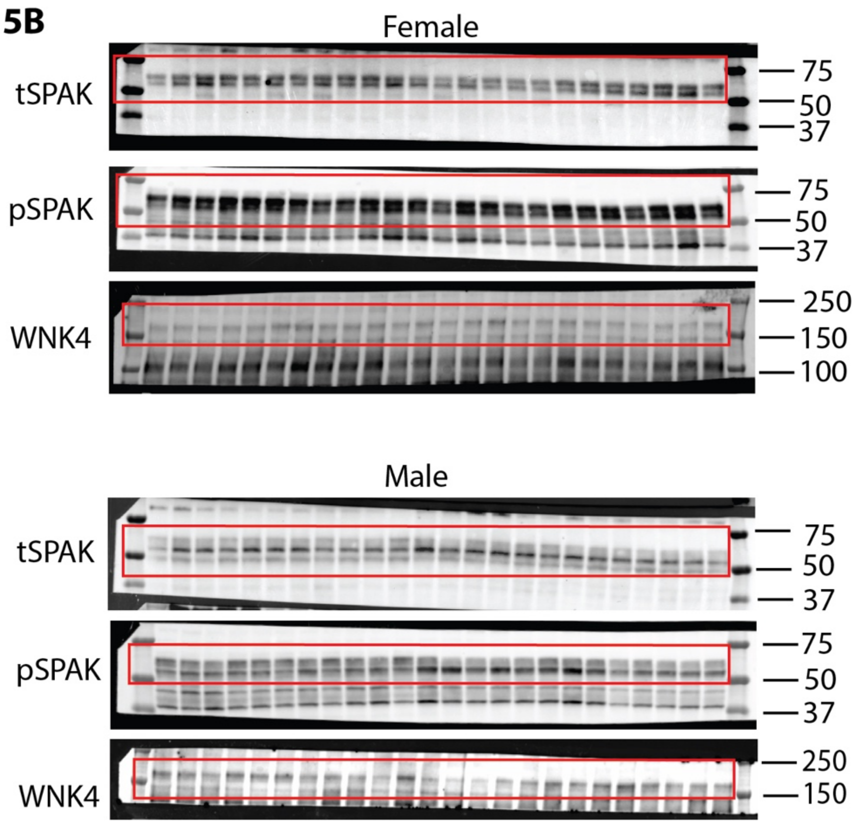

Fig 5B tSPAK is duplicated in Fig S6A blot #2 female

Fig 5B pSPAK is duplicated in Fig S6C blot #5 female

Fig 5B WNK4 is duplicated in Fig S6E blot #8 female

Fig 5B tSPAK is duplicated in Fig S6A blot #2 male

Fig 5B pSPAK is duplicated in Fig S6C blot #5 male

Fig 5B WNK4 is duplicated in Fig S6C blot #8 male

Figure 5C Uncropped Blots

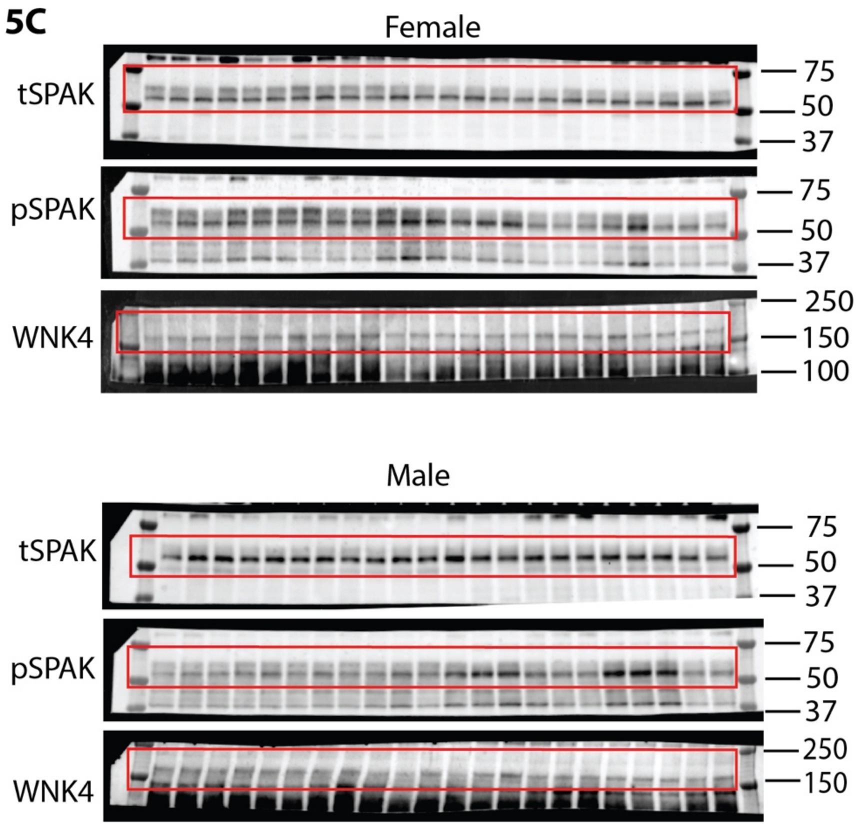

Fig 5C tSPAK is duplicated in Fig S6A blot #3 female

Fig 5C pSPAK is duplicated in Fig S6C blot #6 female

Fig 5C WNK4 is duplicated in Fig S6E blot #9 female

Fig 5C tSPAK is duplicated in Fig S6A blot #3 male

Fig 5C pSPAK is duplicated in Fig S6C blot #6 male

Fig 5C WNK4 is duplicated in Fig S6C blot #9 male

Figure 10A Uncropped Blots

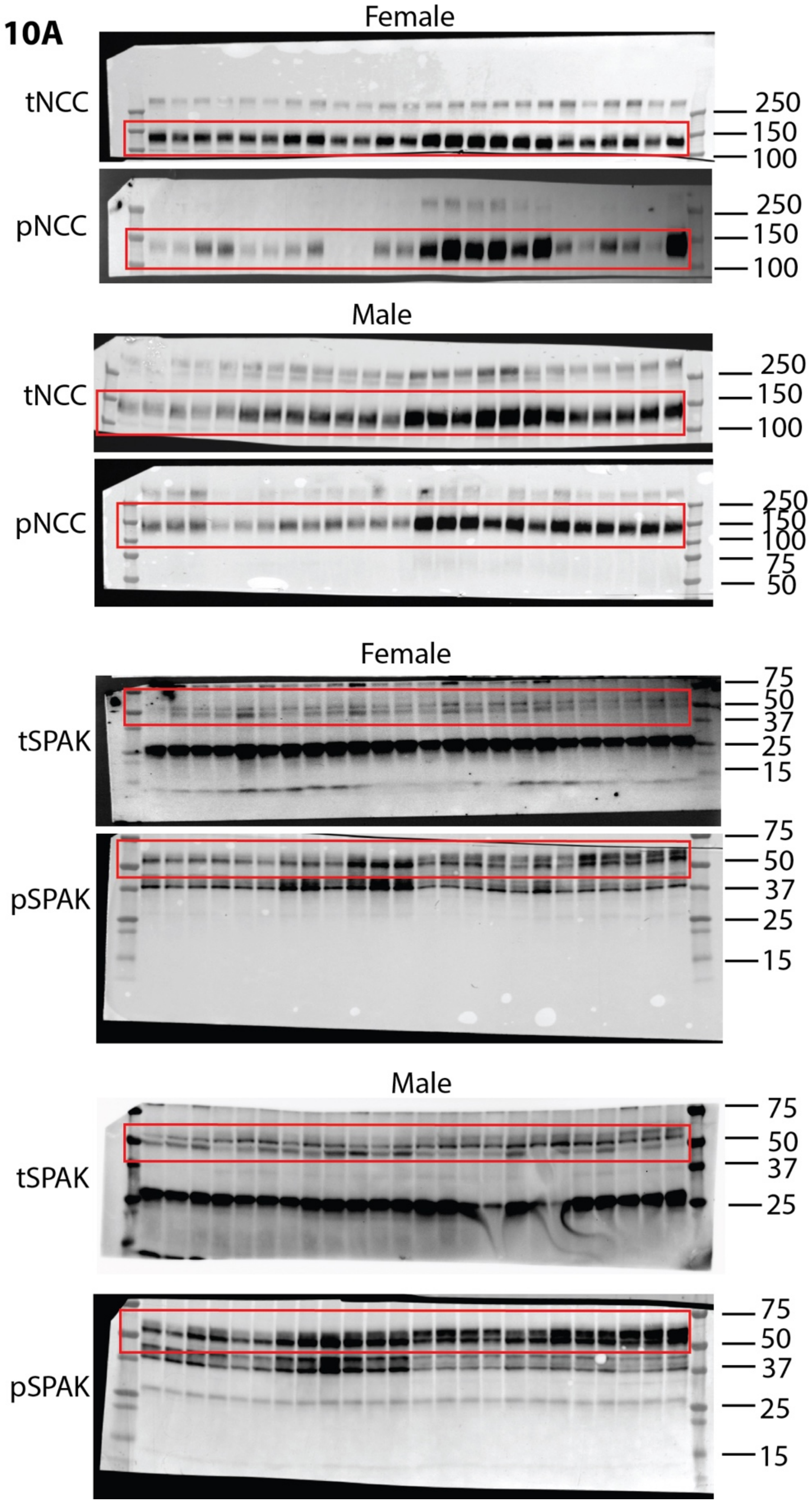

Figure S3 Uncropped Blots

S3A

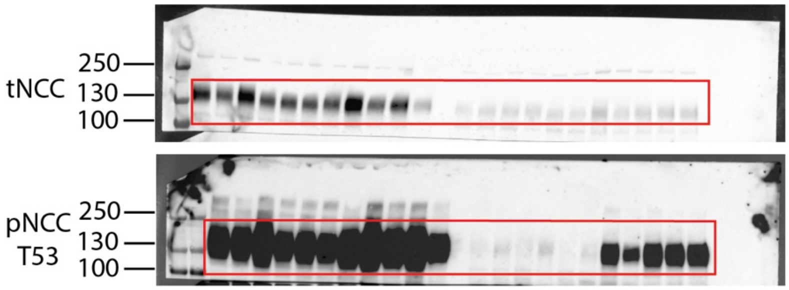

Fig S3A is duplicate of Fig 2C

S3B

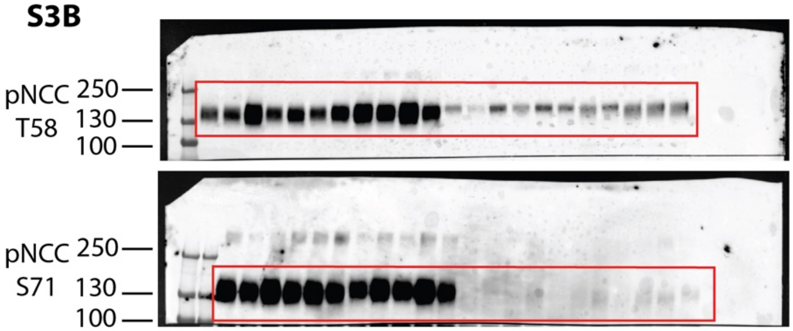

S3C

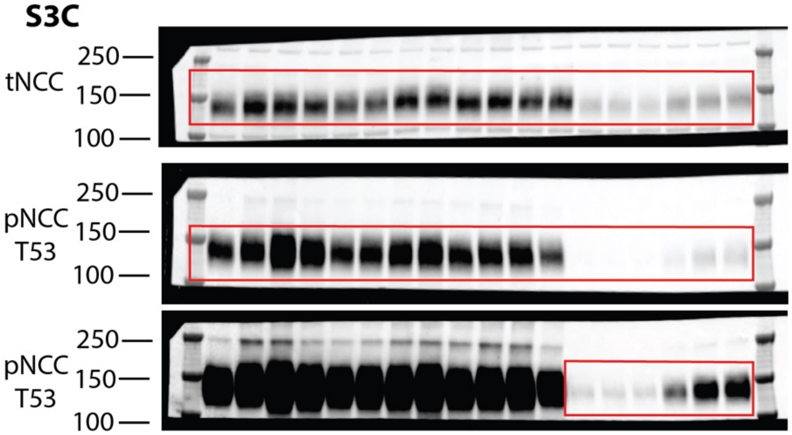

S3D

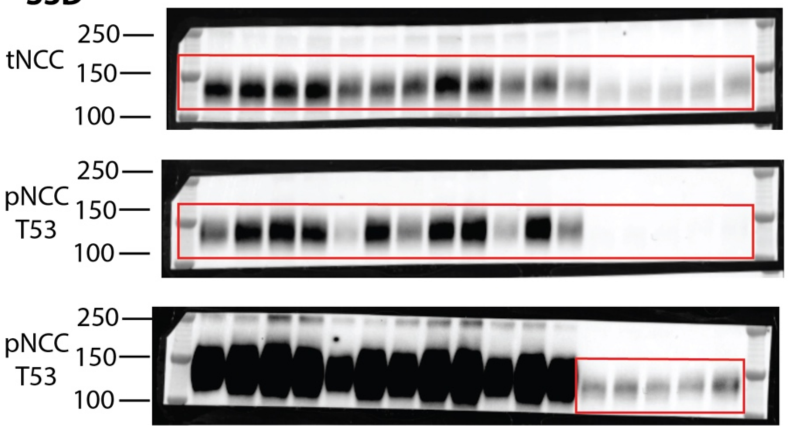

Figure S4 Uncropped Blots

S4A

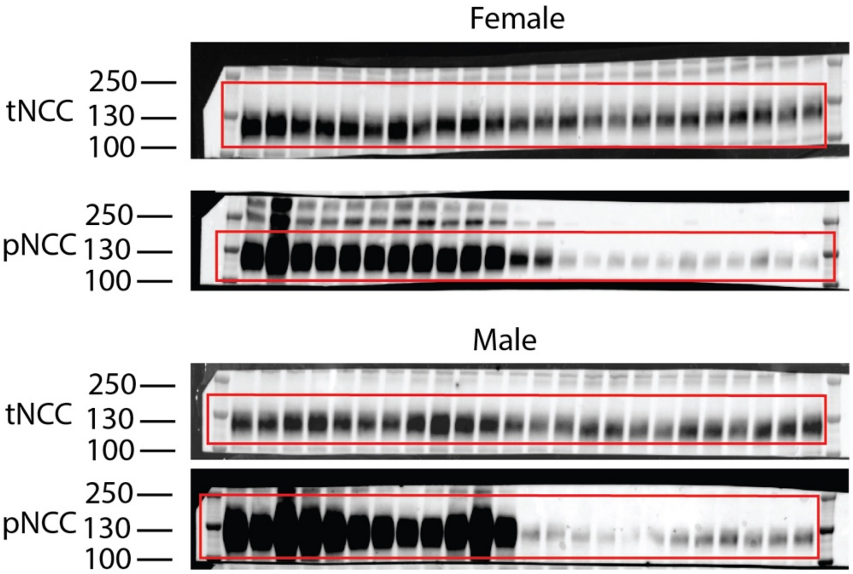

Fig S4A tNCC is duplicated in Fig S2A blot #4

Fig S4A pNCC is duplicated in Fig S2C blot #8

Fig S4A tNCC is duplicated in Fig S2E blot #12

Fig S4A pNCC is duplicated in Fig S2E blot #16

Figure S7 Uncropped Blots

S7A

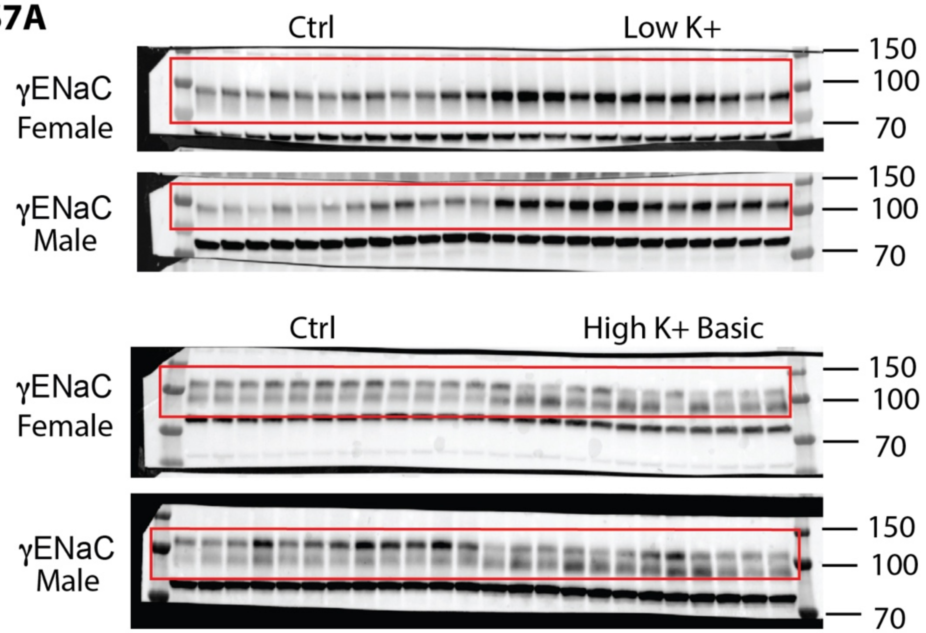

S7D

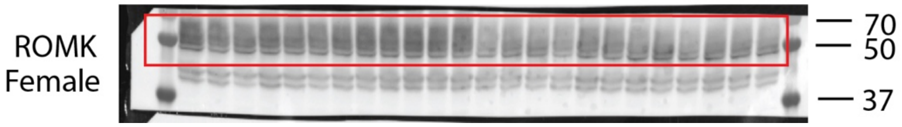

Supplement: Unedited blot and gel images [file jci-135-188792-s110.pdf]
